# Global data-driven predictions of seasonal non-tectonic signals in vertical GNSS displacement time series from non-tidal surface loading data

**DOI:** 10.1186/s40623-026-02385-z

**Published:** 2026-02-19

**Authors:** Kaan Çökerim, Henryk Dobslaw, Kyriakos Balidakis, Laura Jensen, Carlos Peña, Jonathan Bedford

**Affiliations:** 1 Tectonic Geodesy Working Group, Institute of Geosciences, Ruhr University Bochum, Bochum, Germany; 2 Section 1.3. Earth System Modeling, GFZ Helmholtz Centre for Geosciences, Potsdam, Germany; 3 Federal Agency for Cartography and Geodesy (BKG), Frankfurt am Main, Germany; 4 University of Potsdam, Potsdam, Germany

**Keywords:** Non-tidal loading, GNSS, Deep learning, Non-tectonic displacements, Geophysical signal separation

## Abstract

Daily displacement time series from Global Navigation Satellite Systems (GNSS) are frequently used to study deformations of the Earth’s surface due to a wide range of different geophysical processes. The recorded deformations result from tectonic activity or non-tectonic processes like volcanism, groundwater fluctuations and atmospheric loading. In addition, local disturbances of the antenna (e.g., snow cover, thermoelastic effects of the monumentation) and artifacts from GNSS processing (e.g., draconitic signals) are sometimes prominently included in coordinate time series. We use a Temporal Convolution Network (TCN) to predict non-tectonic vertical GNSS displacements on a global scale from physics-based non-tidal loading products. We train our model on a global dataset with more than 11,000 GNSS stations from the Nevada Geodetic Laboratory, active from January 2002 until June 2024, and evaluate the performance against independent estimations. Across the hold-out dataset, our TCN derives non-tidal loading GNSS signatures that when compared to the non-tectonic GNSS signal results in a global average reduction in RMSE of 4.7 % with respect to the numerical non-tidal loading models. This approach presents an initial step towards a data-driven complement to physics-based numerical loading models, improving the isolation of non-tectonic signals in GNSS time series and validation of numerical non-tidal loading models.

**Graphical Abstract:**

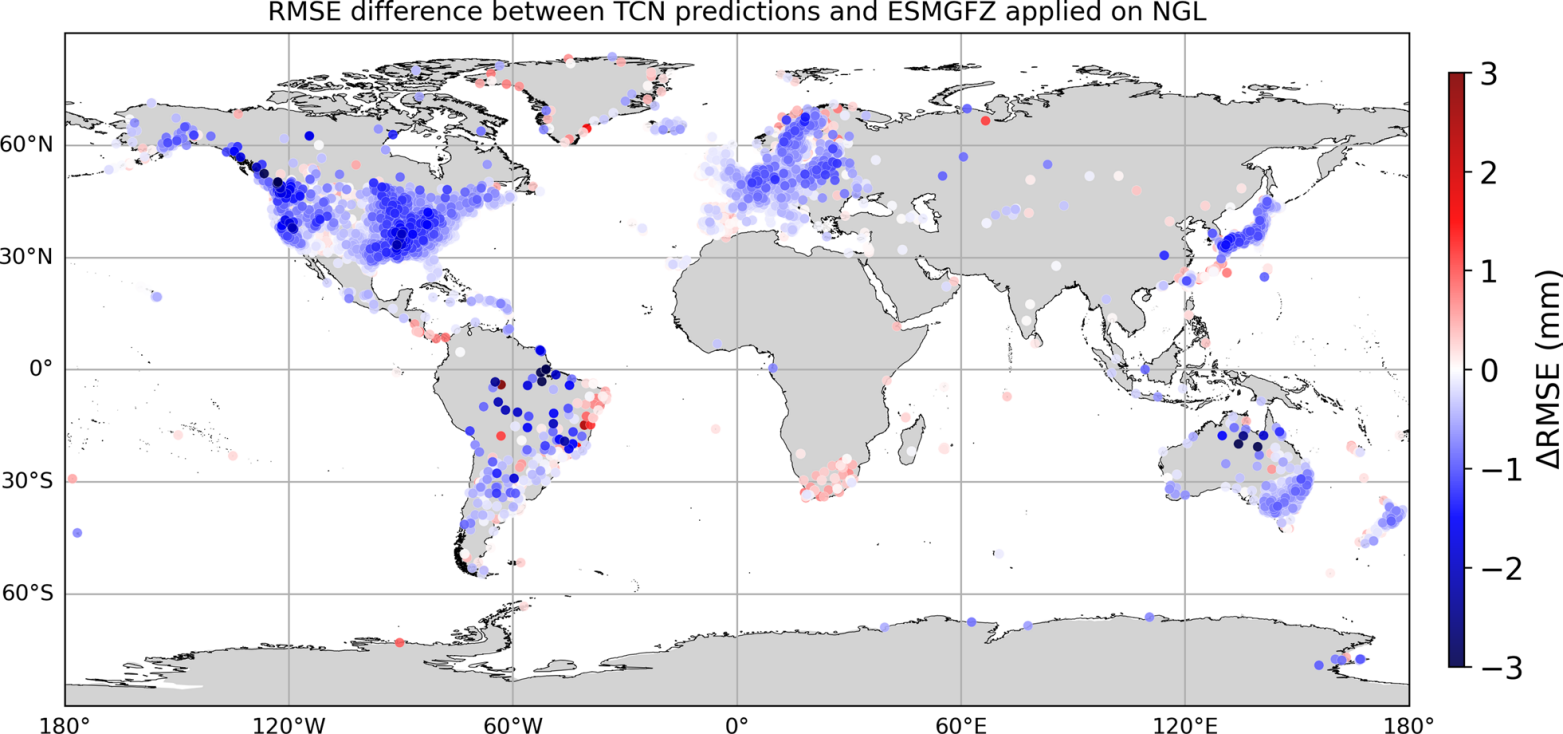

**Supplementary Information:**

The online version contains supplementary material available at 10.1186/s40623-026-02385-z.

## Introduction

Daily displacement time series from Global Navigation Satellite System (GNSS) are frequently applied to study physics and dynamics of the lithosphere at a wide range of spatial and temporal scales. GNSS provides tectonic signals caused by processes such as earthquakes, slow slip and transient events, but also viscoelastic relaxations following large-scale mass shifts (e.g., Wang et al. 2012, Bock and Melgar, 2016, Chanard et al. 2018, Peña et al. 2020, Piña-Valdés et al. 2022). GNSS also captures elastic deformations induced by near-surface mass redistributions (i.e., non-tidal loading) in the atmosphere, oceans (van Dam et al. 1997; Williams and Penna 2011; Gobron et al. 2021), and the terrestrial hydrosphere (e.g., van Dam et al. 2001). Other sources of surface deformations recorded by GNSS are, e.g., viscoelastic deformations related to glacial isostatic adjustment, non-elastic deformations due to volcanic activity, soil compaction due to groundwater withdrawal (Bawden et al. 2001), and other poro- or thermo-elastic processes in the ground (Dong et al. 2002). GNSS time series are therefore of great interest to a wide range of scientists, but the separation of the individual contributions of the various processes is a highly non-trivial task.

Prominent transient signals in GNSS coordinate time series are caused by non-tidal surface loading from fluid mass redistribution in the atmosphere, oceans and terrestrial water storage systems (Davis et al. 2012; Männel et al. 2019; Mémin et al. 2020). While non-tidal atmospheric (NTAL) and oceanic (NTOL) loading were found to cause strong crustal deformation at sub-seasonal periods (van Dam et al. 1994, 1997; Boy and Chao 2005; Williams and Penna 2011; Gobron et al. 2021), it is now established that long-periodic variations are stronger related to hydrological loading (HYDL) (e.g., van Dam et al. 2001, Silverii et al. 2020, Argus et al. 2022). For the isolation of any episodic or even transient tectonic signals, surface loading-related effects need to be precisely predicted and subsequently removed.

Besides geophysical signals, the recorded GNSS times-series also contain systematic error artifacts or site-specific residual noise. The residual noise signals have a particularly high influence on the vertical component due to both the observation geometry (satellites signals are only received from above the station) and various assumptions made at the GNSS processing stage (Gómez et al. 2022). As such, coordinate time series from the same receiver will vary between processing centers. Niu et al. (2022) identified a 10 % discrepancy between the vertical annual signals in GNSS time series from the International GNSS Service (IGS) and NTL models, attributing it to variations in the processing and analysis methods used by the different IGS analysis centers. Consequently, all analysis performed on a set of GNSS displacement solutions will be affected by dataset-specific processing and system noise. The residual noise commonly originates from effects such as multi-path, atmospheric delays, clock drifts and also datum deficits of the global GNSS station network.

Previous attempts to separate noise and geophysical signals were based on Kalman filtering and spectral analysis (e.g., Klos et al. 2018) or independent component analysis (Larochelle et al. 2018; Gualandi and Liu 2021), while others have used harmonic curve fitting as part of trajectory models (Bedford and Bevis 2018; Köhne et al. 2023) and corrections applied already to observation level GNSS time series (e.g., Männel et al. 2023). As described by Bevis et al. (2020), in the application of trajectory models it is commonly assumed that the tectonic and non-tectonic signals are superposed in the total GNSS displacement time series and can be separated. Accordingly, the GNSS displacement signal can be regarded here as a linear combination of basis functions. These basis functions usually consist of a first order polynomial, Heaviside step functions (for earthquakes and hardware and other jumps in the time series), transient functions (such as arctangents, sum of exponentials, B-splines), and sinusoidal oscillations. Although these aforementioned parametric and blind matrix decomposition methods were successful to varying degrees, they do not take into account the underlying physical processes modulating the non-tectonic GNSS displacement signal but instead often make assumptions that simplify its complex signature to basic sinusoidal functions. This approach might cause artifacts such as attributing low-power tectonic signals to the non-tectonic signal because they could not be captured by the respective algorithms or the introduction of artificial transient signals.

Employing machine learning methods to estimate and separate non-tidal loading signatures in GNSS displacement time series has the potential to improve the accuracy of physics-based non-tidal loading models by identifying deformations that are not yet fully captured in numerical models or filtering residual noise signals in GNSS, e.g., artifacts like draconitic errors (Ray et al. 2008; Niu et al. 2022). Recently, various machine learning models have been developed to address current issues in tectonic geodesy including slow slip detection in daily GNSS by Costantino et al. (2023) and earthquake detection by Dittmann et al. (2022) on 5 Hz GNSS displacements. Further, Mastella et al. (2025) developed a neural denoiser to reduce the high-frequency noise in daily GNSS displacement time series. For modeling of the non-tidal loading contributions in GNSS, Ruttner et al. (2022) developed a Temporal Convolutional Network (TCN) to predict residual GNSS displacements in Central Europe from meteorological input features. Their model, however, did not achieve a consistent improvement with respect to the non-tidal loading data. Further, the Ruttner et al. (2022) model, through the choice of meteorological input parameters is strongly bound to atmospheric influences. A global scale, deep learning based assessment of the non-tidal loading signal in GNSS displacement time series has not yet been explored.

We present in this paper a Temporal Convolution Network to predict non-tectonic GNSS displacements based on a globally distributed set of precise-point-positioning (PPP) solutions from the Nevada Geodetic Laboratory (NGL) and the physics-based numerical loading datasets from the Earth System modeling group at GFZ (ESMGFZ) as input features. The TCN architecture is well-suited for time series prediction as it overcomes the limitations of recurrent networks in capturing long-range dependencies. By leveraging stacked dilated convolutions, TCN efficiently model both short- and long-term structures while offering greater computational efficiency than recurrent architectures due to their completely convolutional nature (Yu and Koltun 2016; Lea et al. 2016; Bai et al. 2018; Chen et al. 2020). The intended outcome of our approach is to have a model that can predict non-tectonic displacement signals so that these signals can be subtracted from the total GNSS displacement time series. Our work is motivated by the situation in tectonic research in which the non-tectonic signal obscures the subtle tectonic signals in the GNSS displacement time series (Klos et al. 2018, 2021b).

We introduce the global precise-point-positioning (PPP) GNSS displacement solutions, a priori isolated non-tectonic signals from GNSS displacement time series and the non-tidal loading dataset in Sect. 2. This is followed by the description of the implementation of the TCN model in Section  3. We apply our model on a global scale and explore the performance of the model against a hold-out dataset of at least 2.5 years in Sect. 4 and also present an evaluation of external GNSS data in Section  5. The paper concludes with a brief summary and outlook towards future work.

## GNSS target time series and non-tidal loading input features

### Vertical GNSS displacements from NGL PPP solution

**Fig. 1:**
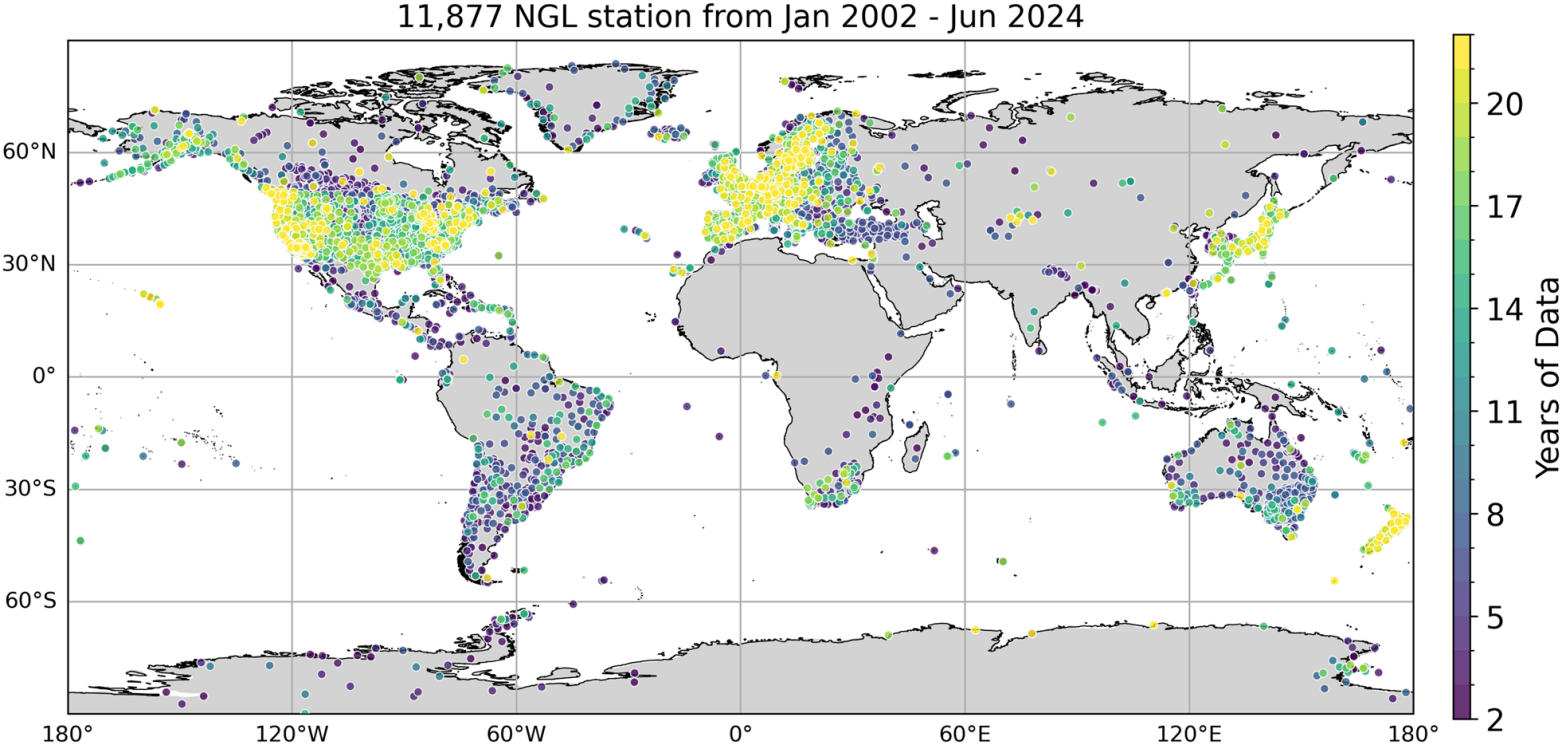
Global distribution of NGL PPP GNSS stations and time series length coverage. Colors denote the available time-series length from a minimum of 2 to 22 years of maximal available recorded time series

For the training of the TCN, we retrieve daily vertical displacement solutions from the Nevada Geodetic Laboratory Blewitt et al. (2018) of 11,877 globally distributed stations that span the period from January 2002 until June 2024. On data retrieval, we impose that the station time series have at least 2 years of recorded data and have a degree of completeness $\ge 85\%$ with a maximum gap length of 60 days. Stations that have multiple segments that fulfill our selection criteria but are separated by gaps $> 60$ days are divided accordingly into segments around these gaps and are treated subsequently as independent stations. The stations in our dataset and their time series lengths are depicted below in Fig. 1a. The station coverage is densest in North and South America, Europe, and Japan, while not many daily displacement time series exist in the NGL database for Africa, Central Asia, Siberia, and China. This creates a spatial bias in the model from the lack of information in certain regions and can only be mitigated when more stations will be available in the future in the sparsely covered regions that can be integrated into the model.

As the target of our deep learning model is going to be the non-tectonic signal of the GNSS displacement time series, we also need to get an initial estimate of these signals to train the model. This step poses a non-linearity in our problem set up in so far that we need an initial algorithm to separate the tectonic and non-tectonic constituents of the GNSS signal in order to train a model that predicts the non-tectonic signal.

To separate and obtain an initial, a priori dataset of non-tectonic and tectonic time series, we perform trajectory modeling using the curve fitting algorithm Greedy Automatic Signal Decomposition (GrAtSiD) by (Bedford and Bevis 2018). Through trajectory modeling, we obtain the non-tectonic signal that contains seasonal effects linked to non-tidal surface loading. Additionally, we capture the residual noise, which may originate from regular system noise or other environmental signals. Such environmental signals include artifacts from thermal expansion of the instrument, monument instabilities, atmospheric signal refraction, and residual ocean tide displacement signals. Furthermore, other artifacts may result from error propagation due to the inherent accuracy limitations from atmospheric and orbit corrections applied during the earlier observation level processing of the GNSS time series as well as multi-path effects, poorly modeled satellite orbit corrections and atmospheric delays (e.g., Mémin et al. 2020, Klos et al. 2021b, Ruttner et al. 2022, Männel et al. 2023). Significant non-linear deformation signals that may originate from processes like glacial isostatic adjustment, volcanic activity, groundwater fluctuations, changes in ice coverage or thermoelastic effects are captured by GrAtSiD as part of the trend which we subsequently remove to obtain the non-tectonic signal. Before applying the trajectory model, we remove outliers in the GNSS displacement times series using a Hampel filter.

We fit the trajectory model with loose constraints to prevent overfitting considering the global scale of our dataset.

After curve fitting with GrAtSiD, we subtract the tectonic constituents (i.e., linear trend, polynomial and transient functions, and known and fitted steps) to leave behind the seasonal oscillations and noise signals. We consider the sum of the seasonal oscillations and noise as the non-tectonic signal and as such as an a priori estimate and target to our machine learning model. In the following sections, we will refer to the decomposed NGL displacement time series simply as as the NGL dataset.

### Global non-tidal surface loading grids from ESMGFZ

The main input features to our model are the vertical surface displacements induced by non-tidal surface loading. We use the global dataset computed and provided by the Earth System Modeling group at the German Research Centre for Geosciences (ESMGFZ) Dill and Dobslaw (2013). The non-tidal surface loading is computed as the elastic response of the Earth’s crust to mass variations obtained from a global numerical land surface scheme in conjunction with a patched Green’s function approach Dill and Dobslaw (2013). The non-tidal loading products are provided on a global $0.5^{\circ }\times 0.5^{\circ }$ grid and consist of the non-tidal atmospheric loading (NTAL), the non-tidal oceanic loading (NTOL)—both provided at 3-hourly sampling—and terrestrial water storage loading (HYDL) at daily, 24-hourly sampling. ESMGFZ further provides the barystatic sea-level variations (SLEL). These are necessary to achieve mass conservation between the oceanic, atmospheric and hydrological loading as the individual models do not consider exchanges of masses between the three domains. However, as described by Klos et al. (2021a), the contribution of SLEL to the total loading signal is low for seasonal and sub-seasonal seasonal signals. As such, we do not consider the sea-level variations as inputs for our modeling.

To obtain the input time series features of all three loading components, we first compute daily averages of the 3-hourly NTAL and NTOL series. Subsequently, we interpolate the non-tidal loading time series spatially at each individual station location. We utilize here the interpolation routine provided by ESMGFZ (https://rz-vm480.gfz.de/repository/entry/show?entryid=8cbb0258-4060-442e-b95f-1fe472f5d142), that uses bi-cubic interpolation, but also implements a land–ocean mask that avoids interpolation over coastlines to prevent mixing of the different deformation responses between continental and oceanic regimes. To avoid interpolation across coastlines, nearest-neighbor interpolation is used for stations that are located along the coast with respect to the resolution of the non-tidal loading grids. The interpolated non-tidal loading time series at each station represent the main input features to our deep learning model associated with their respective non-tectonic target time series.

The sum of the non-tidal loading products is often used as physics-based baseline model to correct the seasonal non-tectonic part of the GNSS signal. Thus, we assume the existence of a mapping function that translates the NTL to the vertical ground displacement recorded by GNSS stations and which we aim to investigate with our deep learning approach. In the following sections, we will refer to the sum of ESMGFZ non-tidal loading products as the "ESMGFZ NTL" or simply ESMGFZ model.

We decided to use non-tidal loading data as opposed to meteorological data like in the study by Ruttner et al. (2022), for two main reasons. Firstly, the amount of different meteorological data that may be relevant is large (e.g., temperature, humidity, precipitation, radiation, surface and ocean bottom pressure and many others) which leaves a huge data space that needs to be coherently explored and tested for redundancy as well as regional dependencies. These data are usually drawn from data repositories like the European Centre for Medium Range Weather Forecast (e.g., Mémin et al. 2020, Ruttner et al. 2022). Secondly, the ESMGFZ non-tidal loading products are generated using these varied streams of meteorological and environmental data and models which are combined with space gravimetric measurements (Dill and Dobslaw 2013). Thus, the numerical non-tidal loading models contain these meteorological and environmental data convolved with with global surface mass observation. Hence, we do not see any benefit in including further meteorological datasets to predict our target.

## Modeling framework and methods

### Deep learning modeling approach and data pipeline

**Fig. 2:**
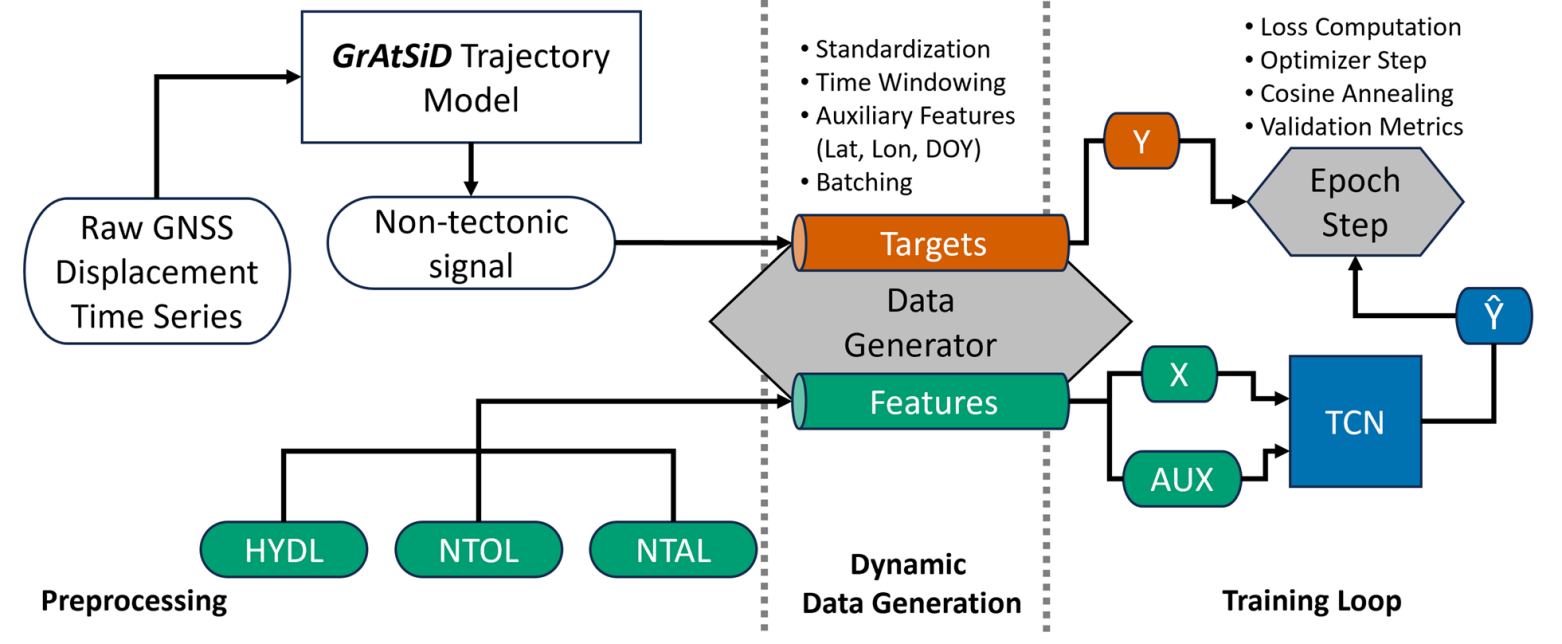
Deep learning workflow of the TCN model. Sketch of the deep learning pipeline including preprocessing, dynamic data tensor generation and training loop

We apply a supervised deep learning model that uses spatially interpolated NTL time series at GNSS station locations as multivariate input features and static auxiliary inputs in the form of station locations, and day-of-the-year to predict the vertical non-tectonic GNSS displacement as the target of our model. We obtain an a priori dataset of vertical non-tectonic GNSS displacement time series through the trajectory modeling with GrAtSiD (see Sect. 2). Our model uses a Temporal Convolutional Network architecture, which utilizes dilated 1D-convolutions to capture and learn temporal patterns of the input features to predict the target. The full data pipeline is illustrated in Fig. 2.

Our model is set up as a many-to-one problem where each training sample consists of three multivariate time series and auxiliary features as input features to the model that predict a single time element in the target sample. The training samples are pooled from a global dataset of GNSS and NTL time series. The input tensor *X* to our TCN model consists of the three multivariate non-tidal loading time series NTAL, NTOL and HYDL at each station. The target *Y* for our model is the non-tectonic signal that we isolated previously through fitting a trajectory model and removing the polynomials, steps and assumed transient tectonic signals. Here, we are essentially trying to find a higher dimensional mapping or transformation between the data spaces of the non-tidal loading and non-tectonic GNSS datasets.

We create input samples using a window length of 365 days for the NTL features to predict a single element of the non-tectonic GNSS signal at time $t_p$. We perform predictions in an acausal setting with look-ahead features such that $X(t\in [t_{p-182}, t_{p+182}])\rightarrow Y(t_p)$, where the predicted sample at $t_p$ is encapsulated by 182 days of feature data in the past and future, respectively. We also tested the causal implementation of the TCN, i.e., using a window that extends 364 days into the past from the prediction time such that $X(t\in [t_{p-364}, t_{p-1}])\rightarrow Y(t_p)$. The causal model did, however, not prove successful. We discuss the difference in performance of the causal and acausal model in detail in Sect. 4.

In addition to the multivariate time series input features, we also provide auxiliary, static input features of the predicted sample $t_p$ at the respective station. One auxiliary feature is the day-of-year of $t_p$ encoded as its sine ($\sin (2\pi t_{p}/365.25)$) and cosine ($\cos (2\pi t_{p}/365.25)$). Further, we provide the latitude encoded by its sine ($\sin (\lambda )$) and the longitude encoded by its sine ($\sin (\phi )$) and cosine ($\cos (\phi )$). Using the trigonometric encodings for day-of-year and geographic coordinates allows us to obtain unique and smooth encodings of these variables. This prevents the day-of-year variable from obtaining a saw-tooth pattern that would be present when transitioning between years where the last day of the year would have a large number (365 or 366) and the following first day of the next year would be at zero. Likewise, the geographic coordinates would jump at the anti-meridian from -180 to 180 degrees of longitude. Hence using the trigonometric encoding ensures smoothness at variable boundaries and prevents jumps and also follows naturally from the definition of the geographic coordinate system. We assume that supplying the auxiliary features together with the characteristic geographic dependencies of the loading signal results in implied learning of geographic features. In the future, an explicit implementation of the location features could be tested with a message-passing graph neural network to handle inter-station relationships in the spatial domain.

Performing the train–validation–test split of the dataset in the spatial domain is expected to cause spatial information leakage because the globally gridded dataset from which the the NTL input features are interpolated is discretized on a $0.5^{\circ }\times 0.5^{\circ }$ grid. The resulting NTL time series can thus be expected to be very similar for close-by stations especially in densely instrumented regions, e.g., in Japan or Cascadia. Therefore, we split all data in the temporal domain, while also taking care to avoid temporal information leakage. We use the time period from 2002–2017 for training, 2017–2021 for validation and the remaining time period from 2021–2024 as our hold-out test dataset. Following the temporal split over all the 11,877 unique stations for the whole period from 2002–2024 results in 5,826 training stations, 9,808 validation stations and 6,932 hold-out test stations. Even though the training period has fewer stations, considering the longer time span covered by the training period, this split results yields sufficiently many training samples. The reduced number of stations is also expected since the global GNSS network is constantly growing. Consequently, the training dataset will have access to enough data to learn multiple annual patterns, while there also remains enough data in the test dataset to study the performance of the prediction model through more than one annual seasonal cycle.

For the purposes of standardization, we calculate a single standard deviation and mean for each feature and target using the the training dataset. During validation and testing, we use the same means and standard deviations to standardize. Standardization is necessary since our input features originate from different sources and have different characteristic amplitudes and spatio-temporal scales.

We perform the training on a high-performance cluster with two NVIDIA RTX A6000 with 48 GB of RAM each. For training, we use a batch size of 1024 samples that are randomly arranged every epoch from a pool of 18.3 Million training samples and 9.9 Million validation samples. We train our models for 500 epochs with an AdamW optimizer (Loshchilov and Hutter 2019) and cosine annealing learning rate scheduler (Loshchilov and Hutter 2017). The cosine annealing scheduler has the benefit that it periodically decreases and increases the learning rate creating an apparent warm restart of training and allowing the optimizer to depart from potential local minima if it should get stuck in one. The AdamW optimizer offers improvements in generalization compared to the more common Adam (Kingma and Ba 2017) by replacing the $L_2$ regularization with decoupled weight regularization as described by Loshchilov and Hutter (2019). For the optimization process, we utilize a loss function that measures the fit through the mean-squared error (MSE) between the predicted and target values. We further use an early stopping callback that stops the training in case the improvement relative to the validation dataset over 70 subsequent epochs becomes worse or insignificantly small. This saves computational time since small improvements in the model metrics do not significantly increase the model accuracy but instead add to its complexity. Additionally, the early stopping prevents the algorithm of leaving the convex point of the learning curve which would lead to an increase of the model misfit.

### Temporal Convolutional Network architecture

**Fig. 3:**
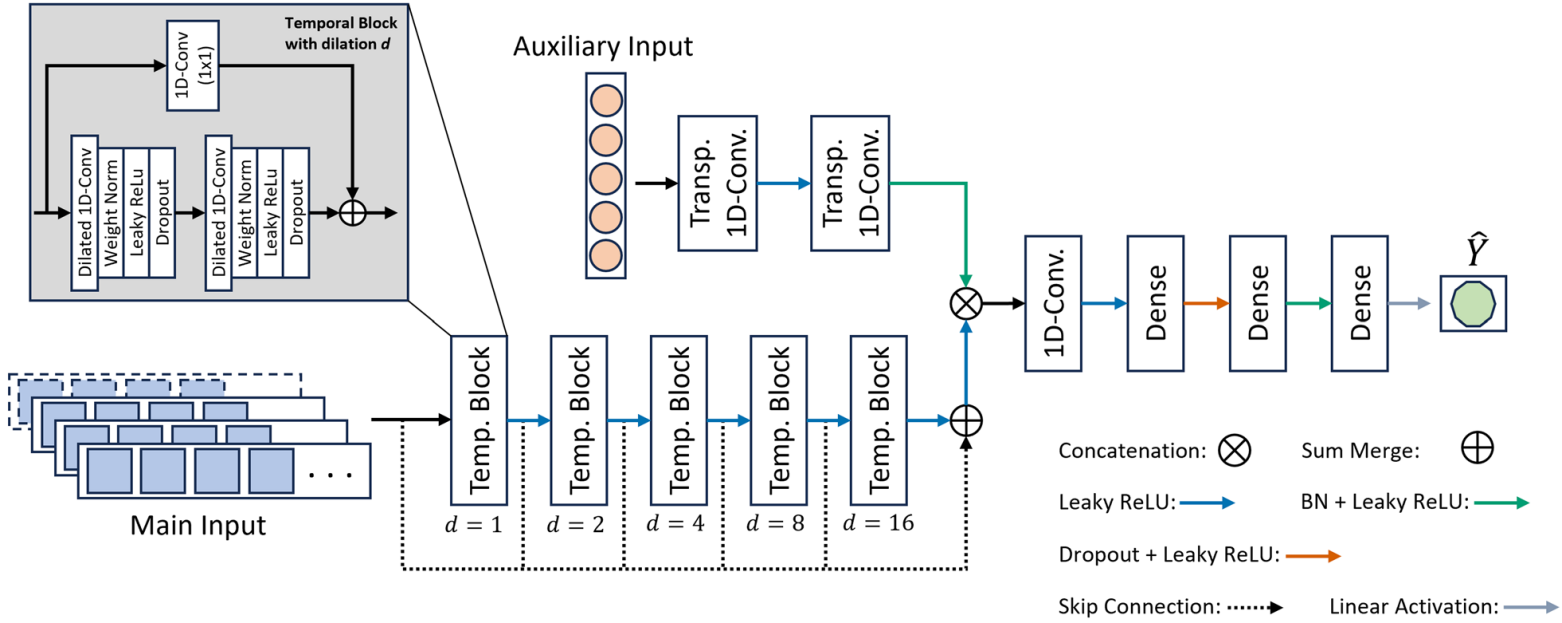
Sketch of our modified Temporal Convolution Network architecture with auxiliary inputs. Concatenation is performed along the channel axis only. The dilation rate is doubled in subsequent temporal blocks. Arrow colors indicate the application of leaky ReLu and linear activation, batch normalization (BN) or dropout layers. Dashed lines indicate skip connections from each temporal block to the end of the stack of temporal blocks. The inset shows a detailed sketch of the temporal block consisting of dilated 1D-convolutions with dilation factor *d* and weight normalization layers. A residual 1D-convolution with 1x1 kernel ensures that the input and output shapes of the temporal block are the same

We employ a Temporal Convolutional Network (TCN) architecture that is commonly used in sequence modeling and time series analysis (Bai et al. 2018; Chen et al. 2020) and which we modify for our specific supervised regression model. The details of our model architecture are illustrated in Fig. 3.

A regular convolution layer assigns each output neuron with the aggregated information within a shifting kernel. The segment information or field from the input layer captured and assigned to a single output neuron is referred to as the receptive field of a convolution layer. The TCN architecture extends this idea by stacking convolutional layers with dilated kernels alternating between active and inactive input segments, which allows to capture information across a larger input width more efficiently by funneling the dilated convolutions into intermediate output neurons in the stack that are then similarly used as inputs to the final convolution layer of the stack (Yu and Koltun 2016). A schematic overview illustrating the concept of the dilated 1D-convolution on a minimal example can be found in the supplementary material in Figure S1. Using zero-padding ensures that the width of the input sequence is preserved in the output sequence. Conservation of shape is necessary because of a later merging operation with a second branch of the network described below. With regard to time series predictions, the convolutions are one-dimensional and each convolution segment of the initial inputs describes a discrete time step in the time series. As such, the dilated convolutions can be used either in a causal or acausal setting, and we are only exploring the latter in this study.

Many recurrent architectures struggle in learning long-range features while also increasing computation time the longer the input time series is. TCN architectures overcome this hurdle through stacked, dilated convolutions (Lea et al. 2016; Bai et al. 2018). As in regular convolutional networks, the convolution layers in TCN further enable the recognition and processing of the general topological structure of the input data, i.e., to account for spatial and temporal context of the input data such as the adjacency of two time steps (LeCun et al. 2015; Bai et al. 2018; Chen et al. 2020). Another benefit is the feature selection capabilities that are inherent to convolution layers and which allow the model to learn meaningful representations of the input (e.g., LeCun et al. 2015). The heart of the TCN architecture is the temporal convolution block which performs the above-mentioned dilated convolutions. The dilated convolution is followed by weight normalization, i.e., normalization by decoupling the magnitude and direction of the weight vectors (Salimans and Kingma 2016; Loshchilov and Hutter 2019), and a dropout layer. The dilation rate is doubled between subsequent temporal blocks to ensure a minimal amount of temporal blocks to increase computation time and prevent the network from getting too deep, creating issues such as vanishing gradients and requiring more training data to prevent overfitting. Our model uses a stack of five temporal blocks with convolution kernel sizes of 16, single stride, and filter sizes of 32 for the first and last two layers while the middle layer has a filter size of 64. The dropout layers in all blocks have a dropout rate of 0.1 while the dilation starts at 1 and then doubles from block to block until a final dilation of 16. The temporal dimension is preserved to allow the multi-scale features of the TCN to be fused consistently with the learned expansion of the auxiliary inputs as described below. Further, we adopt a skip-accumulation pathway similar to van den Oord et al. (2016), summing the outputs of the temporal blocks to form a multi-scale representation before fusion with the auxiliary inputs.

In the second branch of the network, we up-sample the auxiliary inputs through two transpose convolutions on one hand to match the shape of the auxiliary inputs with the output of the temporal block stack to allow for concatenation later on and on the other side to obtain a higher dimensional embedding of the auxiliary features that can modulate the learned temporal feature maps from the temporal blocks. We merge the results of the temporal block stack with the auxiliary input branch of the network by concatenation along the channel dimension and subsequent 1D-convolution before feeding the combined tensor into a stack of three fully connected dense layers with a final linear activation function to produce the prediction output $\hat{Y}$. The 1D-convolution after the concatenation fuses the auxiliary and temporal features by allowing the auxiliary inputs to modulate modulate the learned temporal patterns before the final regression. The dense layers successively half the number of features except the last dense layer which outputs a single feature. In our machine learning task, we seek to predict a continuous scalar value from an input that likewise contains a large matrix of continuous scalar values.

## Evaluation of the TCN performance on the NGL hold-out dataset

We assess the performance of the TCN predictions on the 6, 932 globally distributed stations in the NGL hold-out test dataset. We use the root mean squared error (RMSE) between the TCN predictions and the decomposed non-tectonic displacement time series of the NGL hold-out dataset (ground truth) as a metric. We remove stations with anomalous RMSE by constraining the eligible stations RMSE for further analysis to the $3\sigma$ confidence interval of the RMSE distribution between the TCN predictions and the NGL data. After removing stations with outlier RMSE values, we remain with 6, 816 stations.

In this section, we focus solely on the NGL hold-out dataset. In the following Sect. 5, we provide a further analysis using external datasets comprising the decomposed non-tectonic displacement times series from the IGS core repro3 network and a non-tidal loading model modified using the OS LISFLOOD hydrology model. The IGS repro3 and OS LISFLOOD datasets are described together with the set up of the experiment in Supplementary Text S1. Both external datasets are available for 266 IGS core repro3 stations that are also in the NGL hold-out dataset. Including the analysis together with these external datasets provides a rigorous assessment of model robustness by comparing TCN predictions across varying noise profiles, data-processing workflows, and the environmental conditions specific to the IGS repro3 observations and the OS LISFLOOD model. For more details on the used datasets, we refer to Supplementary Text S1.

We benchmark the TCN predictions of the non-tectonic GNSS displacement signal on the 6, 816 NGL hold-out stations against the numerical ESMGFZ NTL model – which is commonly applied to remove the non-tidal loading signals from GNSS time series. For the benchmark, we compare the RMSE between the ESMGFZ NTL and NGL data (refereed to from now on as RMSE(ESMGFZ NTL)) against the RMSE values computed between the TCN predictions and the NGL data (referred to from now on as RMSE(TCN)).

**Fig. 4:**
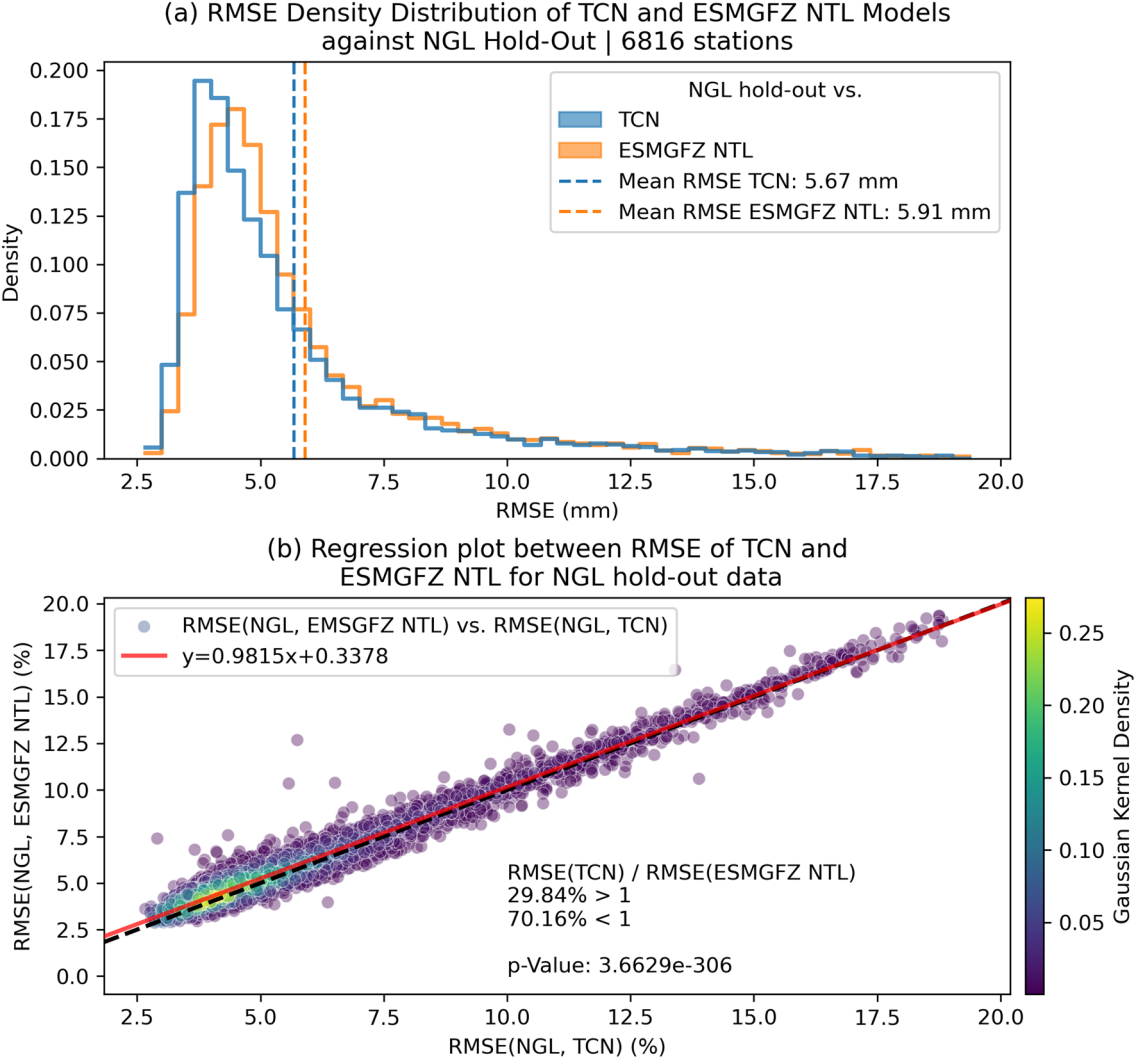
RMSE statistics of the TCN predictions and ESMGFZ NTL model w.r.t. the NGL targets. **a** Density histograms for the RMSE between the non-tectonic NGL hold-out time series and TCN (blue) and ESMGFZ NTL (orange). **b** shows a scatter plot of the RMSE between NGL and TCN on the x-axis and the RMSE between NGL and ESMGFZ NTL. The red line in (**b**) marks a linear region fit through the point cloud with the line equation given in the legend. The black dashed line represents the 1:1 ideal model line. The scatter dots are colored according to statistical density distribution obtained through a gaussian kernel density estimate. In **b**, we additionally label the fraction of data point below the the ideal model line (70.16 %) where the TCN outperforms the ESMGFZ NTL. We also label the *p*-value ($3.67\times 10^{-306}$) calculated using a paired *t*-test between the two RMSE distributions, which indicates strong statistical significance of the TCN performance despite the seemingly small difference in mean RMSE between the TCN and ESMGFZ NTL model

Figure 4a shows the density histogram of the RMSE distribution for both TCN and ESMGFZ. The RMSE(TCN) distribution is shifted more to towards lower values compared to RMSE(ESMGFZ NTL) indicating that the TCN on average achieves lower RMSE value (mean TCN: 5.67 mm) than ESMGFZ NTL (mean: 5.91 mm). The absolute difference between the mean RMSE values is 0.24 mm or −4.7 % improvement of the TCN w.r.t. to the ESMGFZ NTL. The scatter plot in Fig. 4b shows the RMSE values of the ESMGFZ model against the TCN model with the scattered dots each representing one station. Even though the regression line through the point cloud remains close to the ideal model line, we observe that 70.16 % of stations show a lower RMSE for the TCN than for the ESMGFZ NTL model. To assess the statistical significance of the difference between the RMSE values of the TCN and ESMGFZ NTL model, we perform a paired *t*-test. We observe a *p*-value of $3.66\times 10^{-306}$ well below the common 0.05 and 0.0001 significance thresholds. The low *p*-value indicates that the probability for the observed difference between the performance of the TCN and ESMGFZ model being driven by random variation is extremely low and thus the RMSE improvement of the TCN over the ESMGFZ model is statistically significant. Thus, our results demonstrate that the TCN model consistently outperforms the ESMGFZ NTL model on the NGL hold-out dataset and although the absolute RMSE difference is modest with 0.24 mm, the extremely low *p*-value indicates that this improvement is consistent across stations and is unlikely due to random variation such as those caused by residual GNSS errors.

**Fig. 5:**
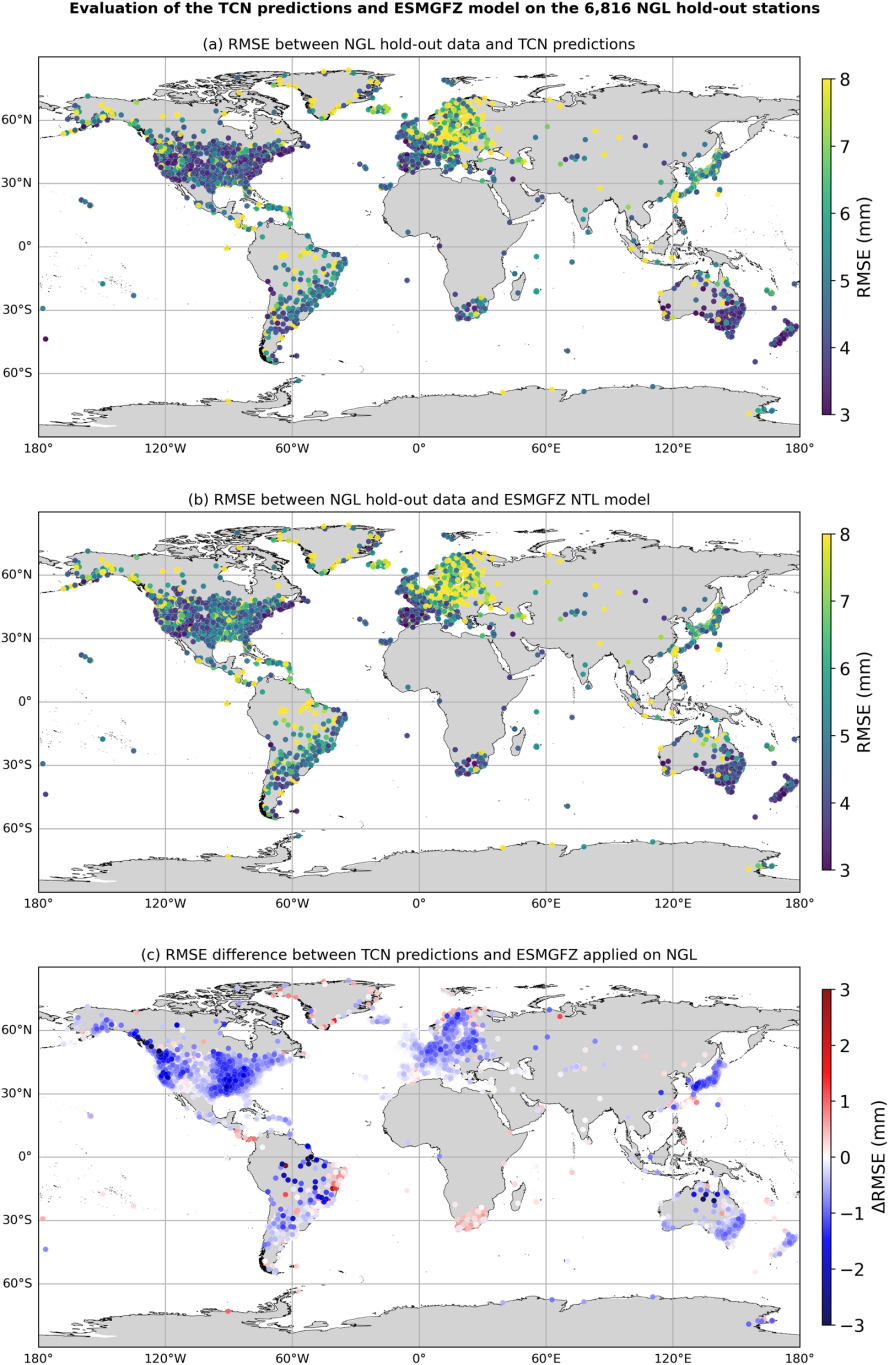
Global RMSE comparison of TCN predictions and ESMGFZ NTL against NGL hold-out stations. Station-by-station RMSE value at the 8816 NGL hold-out stations between non-tectonic NGL time series and **a** TCN predictions and **b** the ESMGFZ NTL model. **c** shows the difference in RMSE between the TCN predictions and ESMGFZ NTL model w.r.t. the NGL hold-out dataset as $\textrm{RMSE}(\textrm{NGL},\textrm{TCN})-\textrm{RMSE}(\textrm{NGL},\mathrm {ESMGFZ\ NTL})$ where negative values (blue) indicate improved performance of the TCN relative to ESMGFZ NTL, while positive values (red) indicate decreased performance

Figure 5a and b shows the RMSE of the TCN and ESMGFZ NTL on a global station-by-station scale. Figure 5c illustrates the difference between RMSE(TCN) and RMSE(ESMGFZ NTL) where blue symbols indicate stations where TCN outperforms the given loading model (lower RMSE), whereas red symbols mark stations where the numeric loading model has a lower RMSE. Most stations indicate a generally lower RMSE for TCN than for ESMGFZ. This aligns with the results from the paired *t*-tests, which showed highly significant improvements of the TCN model. In some regions the ESMGFZ NTL still outperforms the TCN. These areas include among others sub-Saharan Africa, Southern Patagonia, the Brazilian Atlantic coast, the Iberian Peninsula and parts of Eastern Europe, which are either effected by hydrology (e.g., droughts and floods), close to coasts or in areas with low amount of training station, suggesting that the performance gap varies with regional geophysical conditions, data quality and station coverage.

From our results in Fig. 5, we observe no clear latitudinal or longitudinal trend in RMSE changes, contrasting the known latitude dependence of non-tidal loading magnitudes (e.g., Dill and Dobslaw, 2013, Mémin et al. 2020, Gobron et al. 2021). We attribute this to the TCN making use of extensive station data and auxiliary geographic features, which enable it to learn and adapt to global spatial patterns.

**Fig. 6:**
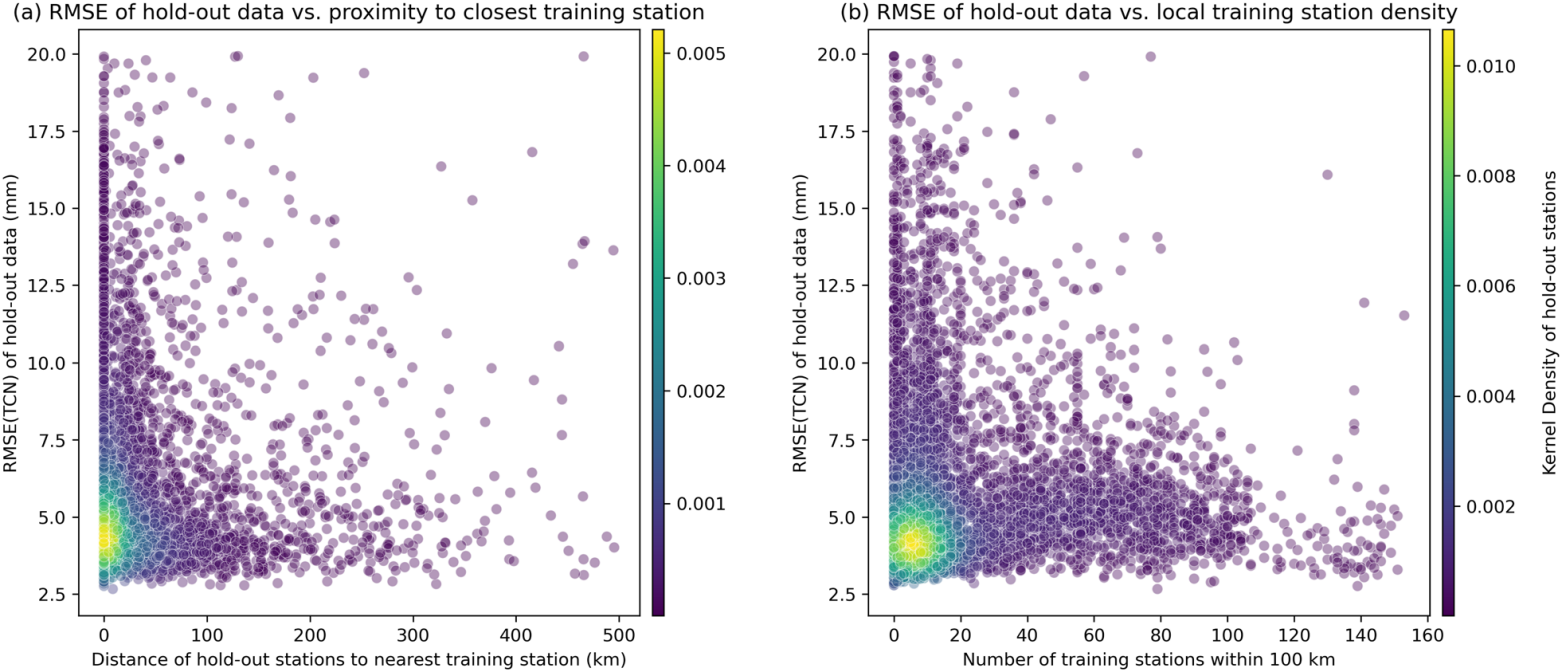
Proximity and neighborhood dependence of the NGL hold-out dataset on the NGL training station coverage. **a** Shows the RMSE(TCN) of each hold-out station against the distance to the closest training station. **b** Shows also the RMSE(TCN) of each hold-out station against the number of training stations that are with a 100 km radius. Colors represent the gaussian kernel density of the distribution for each station point

In Fig. 6, we investigate the influence of proximity to training stations on the TCN model predictions made for the stations in the NGL hold-out dataset. We show the RMSE values of the TCN prediction against the distance to the nearest training station (Fig. 6a) and the number of training stations within 100 km (Fig. 6b) together with the gaussian kernel density to highlight the prominence and clustering of data samples. We observe that TCN errors fall from above 10 mm at remote locations and flattens out in band between 3-6 mm as soon as a station lies within approximately 50 km of one training site or has just 5–10 neighbors. Adding more stations yields only marginal further decrease of the RMSE. The presence of some large RMSE values even at small station separations reflects site-specific signals and data-quality issues that are not represented in the NTL inputs, so geometric proximity to a training station does not necessarily imply similar predictability. Together, this analysis shows that minimal but temporally continuous local coverage during training is sufficient for the TCN to perform well, while areas with denser training coverage are where the TCN delivers its greatest advantage over the numerical loading model.

**Fig. 7:**
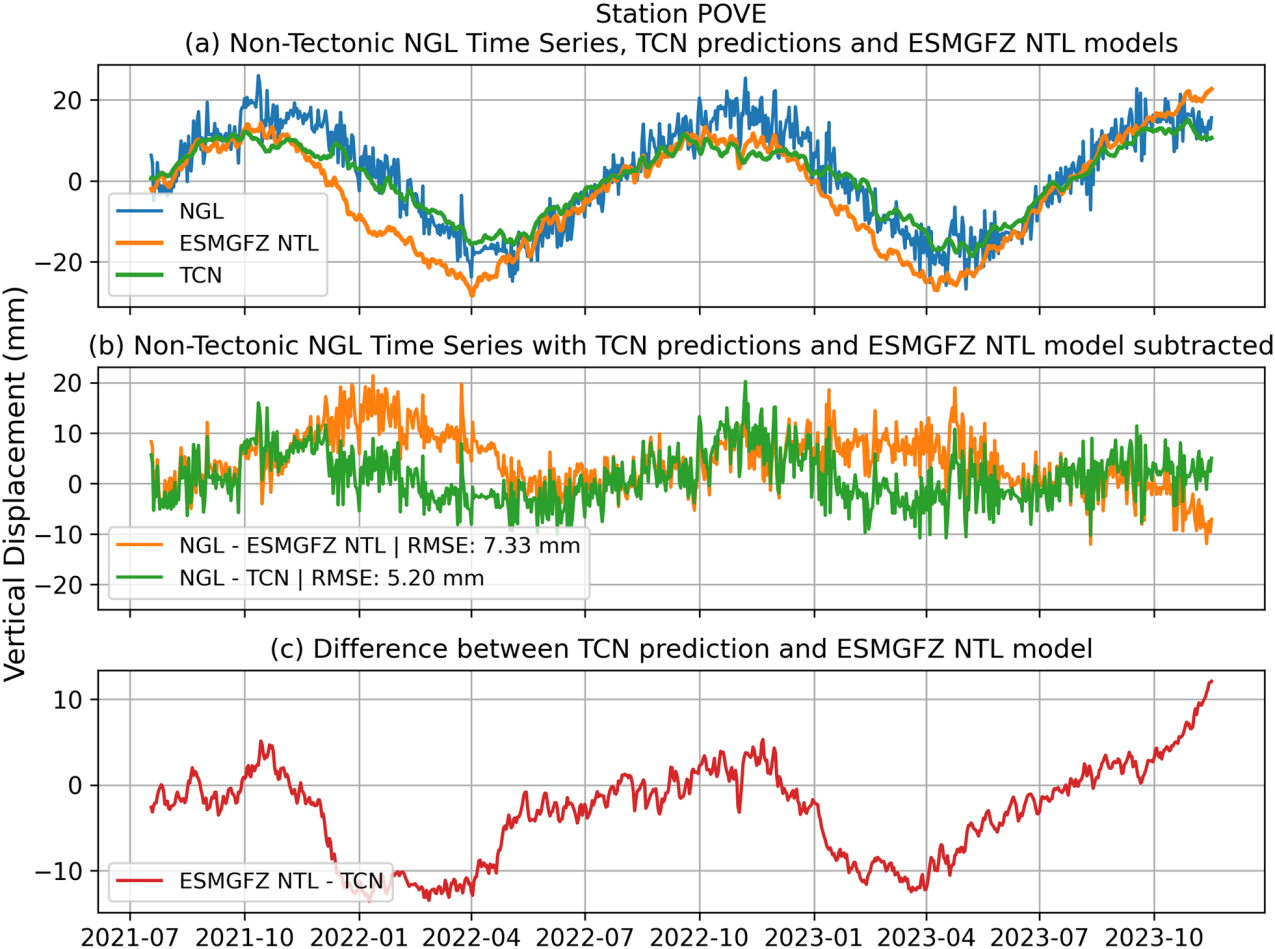
TCN predictions at station POVE and comparison to ESMGFZ NTL and NGL target time series. **a** Decomposed non-tectonic GNSS displacement time series at station POVE (Porto Velho, Rondônia, Brazil) from the NGL (blue) along with model predictions from the TCN (green), ESMGFZ NTL (orange). **b** Shows the residual of TCN subtracted from NGL (green) and ESMGFZ NTL subtracted from NGL (orange). **c** Highlights the difference between ESMGFZ NTL and TCN

**Fig. 8:**
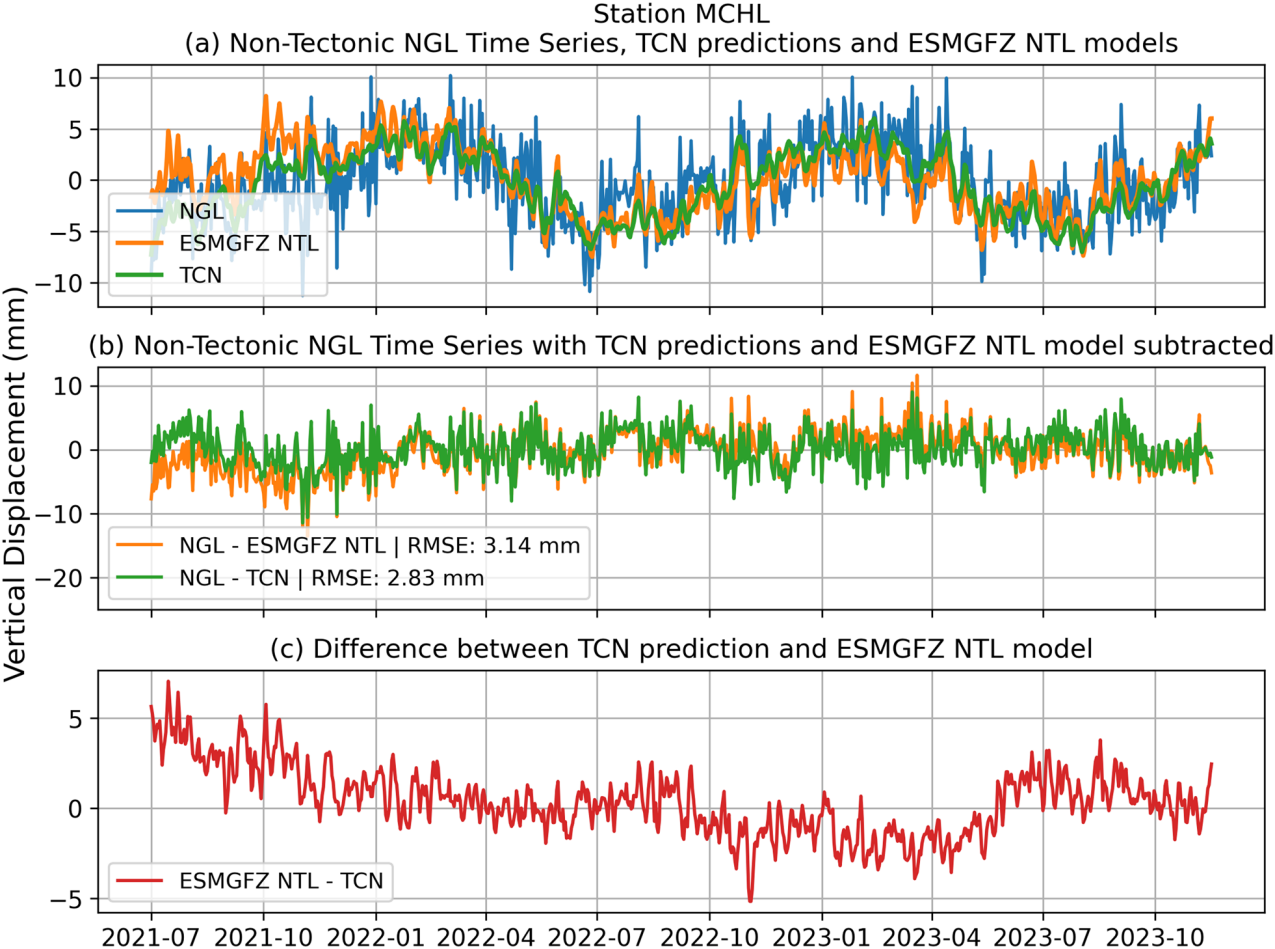
TCN predictions at station MCHL and comparison to ESMGFZ NTL and NGL target time series. **a** Decomposed non-tectonic GNSS displacement time series at station MCHL (Walhallow, Queensland, Australia) from the NGL (blue) along with model predictions from the TCN (green), ESMGFZ NTL (orange). **b** Shows the residual of TCN subtracted from NGL (green) and ESMGFZ NTL subtracted from NGL (orange). **c** Highlights the difference between ESMGFZ NTL and TCN

Figures 7 and 8 display the decomposed non-tectonic displacement time series of the NGL hold-out dataset together with the TCN predictions and ESMGFZ NTL time series at the stations POVE (Porto Velho, Brazil) and MCHL (Walhallow, Queensland, Australia) with both time series exhibiting continuous time series from July 2021 till December 2023.

At POVE in Fig. 7a exhibits a strong seasonal oscillation. The ESMGFZ model consistently underestimates peak amplitudes and lags behind the NGL signal—particularly during southern-hemisphere summer peaks (November–January) and winter troughs (April–July). In contrast, the TCN closely reproduces both phase and amplitude. This is highlighted by the residual time series between NGL and TCN predictions in Fig. 7b yielding a flatter sequence compared to the residual between NGL and ESMGFZ. The residual between the TCN predictions and ESMGFZ NTL time series in Fig. 7c further pronounces the differences of TCN and ESMGFZ NTL by showing a significant deviation of the two models between the months of January and April. While the ESMGFZ yields a RMSE of 7.33 mm the TCN achieves a 29.06 % improvement with a RMSE of 5.20 mm. Given the stations location in the Amazon river basin, the results might highlight issues in the ESMGFZ models hydrological component in accurately modeling the seasonal loads during the summers with increased water levels in this area while the water storage phases during the southern hemisphere winter are modeled quiet well. This might also be caused by real-time changes in climate and weather patterns that are not captured by the numerical ESMGFZ model. Such climate related events are bound to effect the non-tectonic seasonal oscillations observed at GNSS station (e.g., Gobron et al. 2021) and might thus vary relative to the numerical models. Generally, we observe at POVE that the TCN learns both the correct amplitude and timing much more precisely than the purely physical ESMGFZ model. This demonstrates the ability of the TCN to adapt to station-specific characteristics (e.g., local hydrology) that numerical loading models may miss.

At the station MCHL (Fig. 8a) located in eastern Australia, the annual cycle is more muted. The ESMGFZ model tends to slightly overshoot the target signal but mainly exhibits a small phase lag relative to the NGL signal. The TCN prediction is again closer in phase and amplitude, yielding a tighter fit to the NGL data. The residuals in Fig. 8b also indicate less variance from the zero line for the TCN model compared to the ESMGFZ. The difference between the ESMGFZ model and the TCN prediction in Fig. 8c shows significant deviation before January 2022 and between November 2022 and May 2023. Besides that the difference is contained to a high-frequency signal mismatch between the two models. These differences might be caused by localized atmospheric effects during these periods. The RMSE decreases from 3.14 mm (ESMGFZ) to 2.83 mm (TCN)—a 9.87 % improvement of the TCN over the ESMGFZ NTL. At MCHL, where the non-tectonic signal amplitude is lower and less variable, both models perform similarly, yet the TCN still yields a measurable improvement. This suggests that the TCN not only excels at large seasonal signals (as at POVE), but also refines predictions of smaller, more subtle seasonal fluctuations.

The TCN prediction on a longer time series extending 5 years at MCHL is illustrated in the supplementary material in Figure S2. The time series is continuous and extends from the validation into the hold-out phase. The figure clearly shows that the model learns and extracts features from the NTL such as the spike in the ESMGFZ NTL time series around October 2022 and maps them to the non-tectonic target signal. This means also that the auxiliary features do not dominate the TCN predictions and that the inter-annual variations in Figure S2 are driven by the NTL input features.

We perform an ablation study to obtain some superficial interpretability of the models inner workings. For this we train three new models which only include two of the NTL input features at a time during training. A plot of example time series at the stations POVE and MCHL is included in the supplementary material (Figure S3 and S4) together with a map showing the global performance of the three ablation study models (Figure S5). The results of the ablation study suggest that NTAL is the most influential features followed by NTOL and lastly HYDL. Unlike NTAL and NTOL, which represent large-scale atmospheric and oceanic loading effects (Williams and Penna 2011; Gobron et al. 2021), respectively, HYDL may introduce spatially variable signals that, while still relevant, e.g., in the Amazon basin (Figure S5), do not generalize as effectively across all stations. As such, HYDL describes more localized loading effects which was also described by e.g., Tregoning et al. (2009) while Mémin et al. (2020) also note the deficiency of current hydrological models to fully represent all deformation processes induced by hydrological loading. These findings underscore the need for improved hydrological models, such as OS LISFLOOD by Jensen et al. (2025) and aims to provide more accurate and comprehensive representations of hydrological variations. Replacing HYDL with advanced hydrological models like OS LISFLOOD in future studies could address these deficiencies and further enhance the ability of machine learning models to isolate non-tidal loading signals effectively.

We further trained a variant of the model with the same set up that instead uses the causal implementation on the TCN. The results of this experiment are illustrated in the supplementary material in Figures S6 and S7. The causal TCN shows globally a mean increase in RMS relative to the acausal TCN of 5.16 %. At the example station POVE we even observe an RMS of 8.13 mm of in contrast to 5.20 mm from the acausal model. The acausal model predictions exhibit a closer alignment with the target time series compared to the causal model predictions. As such we discard the causal implementation. We propose that with our current model set up, the context provided by only past inputs may not be enough in a global setting and requires the use of future time steps to capture the periodic behavior of the target. Nevertheless, our current model results demonstrate the feasibility of the general approach of using the TCN to predict non-tectonic GNSS displacements from non-tidal loading input series on a global scale. One major drawback is that the acausal implementation causes a 182 days latency for applying our model to real-time data. Future iterations of the model implementation and feature engineering could invest into solving the latency issue.

In summary, the TCN consistently outperforms the traditional ESMGFZ NTL model across a global hold-out dataset, achieving modest but statistically significant RMSE reductions. These results demonstrate the model’s robustness to diverse environmental conditions and station coverage, underscoring the potential of machine learning approaches for improving non-tectonic GNSS displacement corrections.

## Evaluation of the TCN performance on the external IGS repro3 dataset

We provide here further analysis involving external datasets, decomposed non-tectonic displacement time series from the IGS core repro3 network and a non-tidal loading model modified with the OS LISFLOOD hydrology model. Both datasets are described in detail in Supplementary Text S1. These external datasets cover 266 stations overlapping with the NGL hold-out set, enabling a robust evaluation of the TCN performance across varying noise profiles, data-processing methods, and distinct environmental conditions. For additional details on the datasets, we refer to Supplementary Text S1. This analysis assesses model robustness by comparing TCN predictions across different noise regimes, processing workflows, and the environmental conditions present in the IGS repro3 and OS LISFLOOD datasets.

To assess the performance and the differences between the IGS repro3 and NGL data, we conduct the evaluation on a subset of stations that bothdatasets have in common. We further apply the same data thresholds and decomposition process that we applied on the NGL data. Our decomposed non-tectonic repro3 dataset contains 266 stations that are also in our NGL dataset. The IGS core repro3 data are commonly assumed to be selected and processed to the highest standards and contain minimal influences from tectonic or noise signals. While that means that the repro3 data should mainly contain non-tectonic signals such as non-tidal loading, they may still contain some tectonic signatures.

We computed the RMSE for the TCN predictions relative to the non-tectonic target signals derived from both the NGL and repro3 datasets. In parallel, we calculated the RMSE for the ESMGFZ model across both GNSS datasets. To assess the statistical significance of the differences in model performance, as well as to evaluate the impact of the underlying dataset characteristics, we conducted paired *t*-tests using a significance level of 0.05. The resulting *p*-values enabled us to quantitatively determine whether the TCN model’s improvements over the ESMGFZ model were robust across the datasets. We conduct the same evaluation using the LISFLOOD-modified NTL as the loading model. The histograms and scatter plots in Figure S8 show that our TCN outperforms the ESMGFZ and LISFLOOD NTL models. The density histogram in Figure S8a shows the distribution of RMSE values for each model. The TCN’s curve is shifted slightly to the left compared to ESMGFZ (mean = 5.34 mm) and LISFLOOD (mean = 5.71 mm), indicating that, on average, the TCN (mean = 5.20 mm) achieves lower RMSE. A paired *t*-test comparing the RMSE values of the TCN and ESMGFZ models yielded statistically significant differences, with *p*-values of $4.5\times 10^{-5}$ for the IGS dataset and $8.7520\times 10^{-21}$ for TCN vs. LISFLOOD (Figure S8b-c). For comparison, a paired *t*-test between the TCN and ESMGFZ model applied to the NGL data on the same set of station yields a *p*-value of $6.8\times 10^{-8}$. These results demonstrate that the TCN model consistently outperforms the traditional ESMGFZ NTL model on both datasets. Although the absolute RMSE difference for the IGS repro3 data is modest (with mean values of 5.20 mm for the TCN versus 5.34 mm for the ESMGFZ model), the extremely low *p*-value indicates that this improvement is consistent across stations and is unlikely due to random variation. Additionally, paired *t*-tests comparing the RMSE values obtained independently from the IGS and NGL datasets revealed significant differences (*p* = $1.9\times 10^{-3}$ for the TCN model and *p* = $8.9\times 10^{-4}$ for the ESMGFZ model).

To visualize these performance contrasts on a global scale, Figure S9 displays the RMSE difference between TCN predictions and the numeric loading models (ESMGFZ and LISFLOOD) for the 266 stations in the IGS repro3 dataset. Blue symbols indicate stations where TCN outperforms the given loading model (lower RMSE), whereas red symbols signify stations where the numeric loading model has a lower RMSE. Most stations indicate a generally lower RMSE for TCN than for ESMGFZ or LISFLOOD. This aligns with the results from the paired *t*-tests, which showed highly significant improvements of the TCN model across the dataset. In certain regions, particularly those dominated by hydrological influences such as India, Sub-Saharan Africa, Argentina, the Iberian Peninsula, and parts of Eastern Europe, the ESMGFZ model but especially the LISFLOOD model outperforms the TCN. When viewed alongside the analogous plots for the NGL dataset (Fig. 5), these maps confirm that the TCN’s performance advantage is robust to differences in data-processing pipelines. The regional clusters of higher or lower improvements are similar across both datasets, reinforcing the conclusion that the TCN model generalizes well beyond its training set. Figure S10 and Figure S11 display the decomposed non-tectonic GNSS signals (from NGL or IGS) at the stations POVE and BADG and compare them with predictions from the TCN and the numeric loading models (ESMGFZ and LISFLOOD). The bottom panels (d) show the difference between the IGS and NGL signals over the same period. A significant difference is visible between the non-tectonic GNSS time series from NGL and IGS repro3 (panels (a) and (d) in Figure S10 and S11). The differences are likely due to the difference in processing by the different analysis centers, i.e., PPP solutions at NGL vs. combined network solutions at IGS. These inherent differences influence the quality of the TCN predictions, since the TCN is trained on the NGL data. However, we observe that the TCN still tracks the non-tectonic signals of the NGL and repro3 more closely than ESMGFZ and LISFLOOD, as also evident by the lower RMSE of the TCN compared to the numerical models and reduced variance from a zero-mean line upon subtracting the TCN from the non-tectonic GNSS time series. These observations underscore the systematic differences noted in our statistical tests. For instance, POVE shows a steady offset in mid-2022, hinting at localized effects or data-processing differences not fully captured by any single loading model. For BADG, the TCN slightly underestimates the peak displacements in late 2021 but otherwise maintains close alignment with the observed time series. Meanwhile, at POVE, the TCN retains a consistently strong fit through multiple seasonal cycles. The numeric loading models show more variability, suggesting they may lack certain station-specific or hydrological nuances captured by the TCN. Panels (b) and (c) highlight the distinction between NGL and IGS observations. While the overall seasonal signals are comparable, some discrepancies in amplitude and residual noise are apparent, reflecting the differing data-processing methods applied by NGL and IGS repro3. Nonetheless, the TCN’s advantage over ESMGFZ and LISFLOOD remains evident in both datasets. Overlaying the geographic station density from the training dataset with the location of stations showing increasing or decreasing RMSE improvements reveals that the TCN model performs best in regions with dense station coverage (approximately five or more stations within $2^{\circ }\times 2^{\circ }$ bins), as illustrated in Supplementary Figures S12 (NGL targets) and S13 (IGS repro3 targets). In contrast, the OS LISFLOOD NTL model is more effective in regions that are underrepresented in the NGL training dataset.

In summary, our analysis shows that the TCN generalizes well to independent GNSS datasets, achieving statistically significant improvements even with modest RMSE reductions (0.14 mm for the IGS repro3 data). Comparisons with the OS LISFLOOD hydrology model highlight the potential of the TCN, particularly when combined with advanced loading models, for reliably capturing subtle non-tectonic signals and aiding in the development of future loading models.

## Summary and conclusion

We present a deep learning model based on a Temporal Convolution Network architecture to predict vertical non-tectonic surface displacements given location, time epoch, and ESMGFZ non-tidal loading products. Our dataset comprised 11,877 globally distributed GNSS stations for which we obtained PPP displacements through the NGL data repositories. We further performed trajectory modeling using GrAtSiD to decompose the GNSS time series and obtain an a priori dataset of non-tectonic displacement time series. These a priori non-tectonic displacement time series are the learning targets of the TCN. We trained the model on the first 19 years of data and use the remaining 3 years of data as hold-out for evaluation. Our input features are non-tidal loading time series derived from the ESMGFZ non-tidal surface loading displacement grids that cover the whole globe and are available throughout all time-epochs considered.

Our evaluation on 6,816 NGL hold-out stations shows that the TCN delivers consistent improvements over the ESMGFZ non-tidal loading model. On average, the TCN reduces RMSE w.r.t. ESMGFZ NTL by 0.24 mm (4.7 %), with 70 % of stations exhibiting lower RMSE for the TCN than ESMGFZ NTL. Global station-by-station evaluation confirm consistently lower RMSE for the TCN, with only a few hydrology-dominated or regions sparsely covered during training favoring ESMGFZ NTL. The TCN model performance presents itself to be related to the geographic station density of the training dataset. A proximity analysis indicates that as few as five training stations within 100 km suffices for the TCN to achieve RMSE values as low as 3 mm.

Major areas with stations that exhibit higher RMSE for TCN than ESMGFZ NTL are mostly outside of the NGL training network. We attribute part of the geographic generalization to the inclusion of the auxiliary geographic input features that help to learn spatial patterns but also to the spatial wavelengths and resolution of the NTL products used for training. Extending current GNSS networks and using an incremental learning approach could be used to fill in the geographic gaps in future iterations of the TCN. Our results demonstrate that a data-driven TCN can reliably enhance non-tectonic GNSS displacement corrections on a global scale, offering a complementary approach to the established numerical loading models and informing future integrations of advanced hydrology inputs.

We trace the robust behavior of the TCN to the training being conducted with large amounts of the noisier NGL PPP solutions as opposed to the rather clean IGS data, which regularizes and stabilizes the model against noise in the target signals. The TCN thus provides improved predictions of loading signatures in areas of dense station coverage within the training dataset. Outside the geographic training domain, OS LISFLOOD proves to be more preferable than the TCN. We conclude that the TCN trained on a single dataset is capable of distinguishing between processing related and non-tidal loading induced signals, thereby confirming the general applicability of the TCN predictions independently of a specific GNSS processing choice. We note that there is of course still a major impact of dataset bias as is evident, e.g., by the major difference of the order of magnitude between the *p*-value obtained on the NGL and IGS repro3 datasets.

A number of limitations of the mathematical framework applied in this study require future investigation and are beyond the scope of this initial study. The spatial and temporal context in our models is only provided indirectly through the auxiliary input features and encoded by trigonometric functions. Likewise, the interactions and dependence between stations is also not explicitly captured by the TCN. Using a graph network architecture could enable involving the spatio-temporal and inter-station interaction in the model. This would further facilitate a self-regularizing effect in the network based on neighborhood interaction, similar to network solutions in GNSS processing. Utilizing attention mechanisms or transformer networks could help to take a step towards an interpretable model (e.g., Xu et al. 2015) that could be used to investigate and develop the physical concepts behind the mapping of NTL to non-tectonic GNSS displacements and also help to reduce the influence of the noise signal. Further, in the temporal domain, the performance of recurrent networks like bi-directional LSTM that are classically used for sequence modeling could be evaluated against the TCN architecture.

In future work, a deep learning framework such as the TCN shown in this study could be easily extended to make predictions of non-tidal loading displacements in all three directional components. A more extensive testing of model performances for different framing input features and output targets should be performed as well; for example extending the input feature space to include information from surrounding regions (not just the loading interpolated above the GNSS station). Such deep learning based predictions of geophysical fluid loading displacement signals can be deployed to better characterize tectonic transient signals, especially in plate boundary regions where transient tectonic motions are more frequent. They may further prove as an data-driven validation for future hydrodynamic models in regions where no GNSS stations are available.

## Supplementary Information

Supplementary Material 1. In the supplementary document, we include additional figures that support the main text and include a supplementary text describing the external GNSS data from the IGS repro3 campaign (Rebischung et al. 2024) and the OS LISFLOOD model (Jensen et al. 2025) as an alternative for the hydrological non-tidal loading component used in the analysis in Sect. 5.

## Data Availability

The model repository is archived and published as Cökerim (2024) under https://doi.org/10.5281/zenodo.13768181 and is in development on https://github.com/TectonicGeodesy-RUB/NTL-TCN. In the repository we also provide a minimal example dataset subsampled from our compiled global dataset. Due to the size of the OS LISFLOOD data, we provide examples for selected stations and refer to Jensen et al. (2025) for the full dataset. We additionally provide target-prediction sets for the 266 IGS core stations in Supplementary Text S1 and scripts to download and process the NGL and ESMGFZ NTL data. The original GNSS displacement time series are obtained from the Nevada Geodetic Laboratory (Blewitt et al. 2018) and are openly available in the NGL data repository under http://geodesy.unr.edu/index.php (Last accessed for download: 01 June 2024). Additional GNSS time series were composed based on the combined IGS repro3 solution (Rebischung et al. 2024) retrieved from ftp://igs-rf.ign.fr/pub/repro3/. The geophysical non-tidal surface loading products by Dill and Dobslaw (2013) are available at the ESMGFZ data repository https://rz-vm480.gfz.de/ (Last accessed for download: 01 June 2024). The alternative hydrological loading model is developed at ESMGFZ and derived from the OS LISFLOOD hydrological model by Van Der Knijff et al. (2010). The derived loading dataset is published as Jensen et al. (2025). As mentioned above, we provide a minimal sample of the OS LISFLOOD derived loading dataset in our model repository. The GrAtSiD trajectory modeling software by Bedford and Bevis (2018) is available on the GitHub repository of the Tectonic Geodesy group at Ruhr University Bochum on https://github.com/TectonicGeodesy-RUB/Gratsid.

## References

[CR1] Abadi M, Agarwal A, Barham P, Brevdo E, Chen Z, Citro C, Corrado GS, Davis A, Dean J, Devin M, Ghemawat S, Goodfellow I, Harp A, Irving G, Isard M, Jia Y, Jozefowicz R, Kaiser L, Kudlur M, Levenberg J, Mané D, Monga R, Moore S, Murray D, Olah C, Schuster M, Shlens J, Steiner B, Sutskever I, Talwar K, Tucker P, Vanhoucke V, Vasudevan V, Viégas F, Vinyals O, Warden P, Wattenberg M, Wicke M, Yu Y, Zheng X (2015) TensorFlow: large-scale machine learning on heterogeneous systems. 10.5281/zenodo.4724125.

[CR2] Ansel J, Yang E, He H, Gimelshein N, Jain A, Voznesensky M, Bao B, Bell P, Berard D, Burovski E, Chauhan G, Chourdia A, Constable W, Desmaison A, DeVito Z, Ellison E, Feng W, Gong J, Gschwind M, Hirsh B, Huang S, Kalambarkar K, Kirsch L, Lazos M, Lezcano M, Liang Y, Liang J, Lu Y, Luk CK, Maher B, Pan Y, Puhrsch C, Reso M, Saroufim M, Siraichi MY, Suk H, Zhang S, Suo M, Tillet P, Zhao X, Wang E, Zhou K, Zou R, Wang X, Mathews A, Wen W, Chanan G, Wu P, Chintala S (2024) PyTorch 2: faster machine learning through dynamic python bytecode transformation and graph compilation. In: Proceedings of the 29th ACM International Conference on Architectural Support for Programming Languages and Operating Systems, Volume 2. Association for Computing Machinery, New York, NY, USA, ASPLOS ’24, pp 929–947. 10.1145/3620665.3640366

[CR3] Argus DF, Martens HR, Borsa AA, Knappe E, Wiese DN, Alam S, Anderson M, Khatiwada A, Lau N, Peidou A, Swarr M, White AM, Bos MS, Ellmer M, Landerer FW, Gardiner WP (2022) Subsurface water flux in California’s Central Valley and its source watershed from space geodesy. Geophys Res Lett 49(22):e2022GL099583. 10.1029/2022GL099583

[CR4] Bai S, Kolter JZ, Koltun V (2018) An empirical evaluation of generic convolutional and recurrent networks for sequence modeling. Comput Res Repos. 10.48550/arXiv.1803.01271

[CR5] Bawden GW, Thatcher W, Stein RS, Hudnut KW, Peltzer G (2001) Tectonic contraction across Los Angeles after removal of groundwater pumping effects. Nature 412(6849):812–815. 10.1038/35090558 11518964 10.1038/35090558

[CR6] Bedford J, Bevis M (2018) Greedy automatic signal decomposition and its application to daily GPS time series. J Geophys Res Solid Earth 123(8):6992–7003. 10.1029/2017JB014765

[CR7] Bevis M, Bedford J, Caccamise II DJ (2020) The art and science of trajectory modelling. In: Montillet JP, Bos MS (eds) Geodetic time series analysis in earth sciences. Springer International Publishing, Cham, pp 1–27. 10.1007/978-3-030-21718-1_1

[CR8] Blewitt G, Hammond WC, Kreemer C (2018) Harnessing the GPS data explosion for interdisciplinary science. Eos 99(2):e2020943118. 10.1029/2018EO104623

[CR9] Bock Y, Melgar D (2016) Physical applications of GPS geodesy: a review. Rep Prog Phys 79(10):106801. 10.1088/0034-4885/79/10/106801 27552205 10.1088/0034-4885/79/10/106801

[CR10] Boy JP, Chao BF (2005) Precise evaluation of atmospheric loading effects on Earth’s time-variable gravity field. J Geophys Res Solid Earth 110(B8):B08412. 10.1029/2002JB002333

[CR11] Chanard K, Fleitout L, Calais E, Barbot S, Avouac JP (2018) Constraints on transient viscoelastic rheology of the asthenosphere from seasonal deformation. Geophys Res Lett 45(5):2328–2338. 10.1002/2017GL076451

[CR12] Chen Y, Kang Y, Chen Y, Wang Z (2020) Probabilistic forecasting with temporal convolutional neural network. Neurocomputing 399:491–501. 10.1016/j.neucom.2020.03.011

[CR13] Costantino G, Giffard-Roisin S, Radiguet M, Dalla Mura M, Marsan D, Socquet A (2023) Multi-station deep learning on geodetic time series detects slow slip events in Cascadia. Commun Earth Environ 4:435. 10.1038/s43247-023-01107-7

[CR14] Cökerim K (2024) TectonicGeodesy-RUB/NTL-TCN: initial pre-release. Zenodo. 10.5281/zenodo.13768181

[CR15] Davis JL, Wernicke BP, Tamisiea ME (2012) On seasonal signals in geodetic time series. J Geophys Res Solid Earth. 10.1029/2011JB008690

[CR16] Dill R, Dobslaw H (2013) Numerical simulations of global-scale high-resolution hydrological crustal deformations. J Geophys Res Solid Earth 118(9):5008–5017. 10.1002/jgrb.50353

[CR17] Dittmann T, Liu Y, Morton Y, Mencin D (2022) Supervised machine learning of high rate GNSS velocities for earthquake strong motion signals. J Geophys Res Solid Earth 127(11):e2022JB024854. 10.1029/2022JB024854

[CR18] Dong D, Fang P, Bock Y, Cheng MK, Miyazaki S (2002) Anatomy of apparent seasonal variations from GPS-derived site position time series. J Geophys Res Solid Earth 107(B4):ETG 9-1-ETG 9-16. 10.1029/2001JB000573

[CR19] Gobron K, Rebischung P, Van Camp M, Demoulin A, de Viron O (2021) Influence of aperiodic non-tidal atmospheric and oceanic loading deformations on the stochastic properties of global GNSS vertical land motion time series. J Geophys Res Solid Earth 126(9):e2021JB022370. 10.1029/2021JB022370

[CR20] Gualandi A, Liu Z (2021) Variational Bayesian independent component analysis for InSAR displacement time-series with application to Central California, USA. J Geophys Res Solid Earth 126(4):e2020JB020845. 10.1029/2020JB020845

[CR21] Gómez DD, Bevis MG, Caccamise DJ (2022) Maximizing the consistency between regional and global reference frames utilizing inheritance of seasonal displacement parameters. J Geodesy 96:9. 10.1007/s00190-022-01594-0

[CR22] Harris CR, Millman KJ, van der Walt SJ, Gommers R, Virtanen P, Cournapeau D, Wieser E, Taylor J, Berg S, Smith NJ, Kern R, Picus M, Hoyer S, van Kerkwijk MH, Brett M, Haldane A, del Río JF, Wiebe M, Peterson P, Gérard-Marchant P, Sheppard K, Reddy T, Weckesser W, Abbasi H, Gohlke C, Oliphant TE (2020) Array programming with NumPy. Nature 585(7825):357–362. 10.1038/s41586-020-2649-2 32939066 10.1038/s41586-020-2649-2 PMC7759461

[CR23] Hunter JD (2007) Matplotlib: a 2D graphics environment. Comput Sci Eng 9(3):90–95. 10.1109/MCSE.2007.55

[CR24] Jensen L, Dill R, Balidakis K, Grimaldi S, Salamon P, Dobslaw H (2025) Global water storage simulations with the OS LISFLOOD hydrological model for geodetic applications. Geophys J Int 241(3):1840–1852. 10.1093/gji/ggaf129

[CR25] Kingma DP, Ba J (2017) Adam: a method for stochastic optimization, arXiv:1412.6980. 10.48550/arXiv.1412.6980

[CR26] Klos A, Boz M, Bogusz J (2018) Detecting time-varying seasonal signal in GPS position time series with different noise levels. GPS Solut 22(1):21. 10.1007/s10291-017-0686-6

[CR27] Klos A, Dobslaw H, Dill R, Bogusz J (2021a) Identifying the sensitivity of GPS to non-tidal loadings at various time resolutions: examining vertical displacements from continental Eurasia. GPS Solut. 10.1007/s10291-021-01135-w

[CR28] Klos A, Karegar MA, Kusche J, Springer A (2021b) Quantifying noise in daily GPS height time series: harmonic function versus GRACE-assimilating modeling approaches. IEEE Geosci Remote Sens Lett 18(4):627–631. 10.1109/LGRS.2020.2983045

[CR29] Köhne T, Riel B, Simons M (2023) Decomposition and inference of sources through spatiotemporal analysis of network signals: the DISSTANS Python package. Comput Geosci 170:105247. 10.1016/j.cageo.2022.105247

[CR30] Larochelle S, Gualandi A, Chanard K, Avouac JP (2018) Identification and extraction of seasonal geodetic signals due to surface load variations. J Geophys Res Solid Earth 123(12):11031–11047. 10.1029/2018JB016607

[CR31] Lea C, Vidal R, Reiter A, Hager GD (2016) Temporal convolutional networks: a unified approach to action segmentation. In: Hua G, Jégou H (eds) Computer Vision – ECCV 2016 Workshops. Springer International Publishing, Cham, pp 47–54, 10.1007/978-3-319-49409-8_7

[CR32] LeCun Y, Bengio Y, Hinton G (2015) Deep learning. Nature 521:436–444. 10.1038/nature14539 26017442 10.1038/nature14539

[CR33] Loshchilov I, Hutter F (2017) SGDR: stochastic gradient descent with warm restarts. In: International Conference on Learning Representations. 10.48550/arXiv.1608.03983.

[CR34] Loshchilov I, Hutter F (2019) Decoupled weight decay regularization. In: 7th International Conference on Learning Representations, ICLR 2019, New Orleans, Louisiana, May 6 - May 9, 2019, Conference Track Proceedings. 10.48550/arXiv.1711.05101.

[CR35] Mastella G, Bedford J, Corbi F, Funiciello F (2025) Denoising daily displacement GNSS time series using deep neural networks in a near real-time framing: a single-station method. Geophys J Int 242(3):ggaf207. 10.1093/gji/ggaf207

[CR36] McKinney W (2010) Data structures for statistical computing in Python. In: van der Walt S, Millman J (eds) Proceedings of the 9th Python in Science Conference, pp 56–61. 10.25080/Majora-92bf1922-00a

[CR37] Met Office (2015) Cartopy: a cartographic python library with a Matplotlib interface. Exeter, Devon. 10.5281/zenodo.1182735. https://scitools.org.uk/cartopy. Accessed 14 Sept 2024

[CR38] Männel B, Dobslaw H, Dill R, Glaser S, Balidakis K, Thomas M, Schuh H (2019) Correcting surface loading at the observation level: impact on global GNSS and VLBI station networks. J Geodesy 93:2003–2017. 10.1007/s00190-019-01298-y

[CR39] Männel B, Brandt A, Glaser S, Schuh H (2023) correcting non-tidal surface loading in GNSS repro3 and comparison with ITRF2020. In: Freymueller JT, Sánchez L (eds) Gravity, positioning and reference frames. Springer Nature Switzerland, Cham, pp 209–216. 10.1007/1345_2023_207

[CR40] Mémin A, Boy J, Santamaría-Gómez A (2020) Correcting GPS measurements for non-tidal loading. GPS Solut 24(2):45. 10.1007/s10291-020-0959-3

[CR41] Niu Y, Wei N, Li M, Rebischung P, Shi C, Chen G (2022) Quantifying discrepancies in the three-dimensional seasonal variations between IGS station positions and load models. J Geodesy 96(4):31. 10.1007/s00190-022-01618-9

[CR42] Pedregosa F, Varoquaux G, Gramfort A, Michel V, Thirion B, Grisel O, Blondel M, Prettenhofer P, Weiss R, Dubourg V, Vanderplas J, Passos A, Cournapeau D, Brucher M, Perrot M, Duchesnay E (2011) Scikit-learn: machine learning in Python. J Mach Learn Res 12:2825–2830. 10.48550/arXiv.1201.0490.

[CR43] Peña C, Heidbach O, Moreno M, Bedford J, Ziegler M, Tassara A, Oncken O (2020) Impact of power-law rheology on the viscoelastic relaxation pattern and afterslip distribution following the 2010 Mw 8.8 Maule earthquake. Earth Planet Sci Lett 542:116292. 10.1016/j.epsl.2020.116292

[CR44] Piña-Valdés J, Socquet A, Beauval C, Doin MP, D’Agostino N, Shen ZK (2022) 3D GNSS velocity field sheds light on the deformation mechanisms in Europe: effects of the vertical crustal motion on the distribution of seismicity. J Geophys Res Solid Earth 127(6):e2021JB023451. 10.1029/2021JB023451

[CR45] Ray J, Altamimi Z, Collilieux X, van Dam T (2008) Anomalous harmonics in the spectra of GPS position estimates. GPS Solut 12:55–64. 10.1007/s10291-007-0067-7

[CR46] Rebischung P, Altamimi Z, Métivier L, Collilieux X, Gobron K, Chanard K (2024) Analysis of the IGS contribution to ITRF2020. J Geodesy 98(6):49. 10.1007/s00190-024-01870-1

[CR47] Ruttner P, Hohensinn R, D’Aronco S, Wegner JD, Soja B (2022) Modeling of residual GNSS station motions through meteorological data in a machine learning approach. Remote Sens 14(1):17. 10.3390/rs14010017

[CR48] Salimans T, Kingma DP (2016) Weight normalization: a simple reparameterization to accelerate training of deep neural networks. In: Lee D, Sugiyama M, Luxburg U, Guyon I, Garnett R (eds) Advances in neural information processing systems. 10.48550/arXiv.1602.07868

[CR49] Silverii F, Montgomery-Brown EK, Borsa AA, Barbour AJ (2020) Hydrologically induced deformation in Long Valley Caldera and adjacent Sierra Nevada. J Geophys Res Solid Earth 125(5):e2020JB019495. 10.1029/2020JB019495

[CR50] The Pandas Development Team (2020) pandas-dev/pandas: Pandas. Zenodo. 10.5281/zenodo.3509134

[CR51] Tregoning P, Watson C, Ramillien G, McQueen H, Zhang J (2009) Detecting hydrologic deformation using GRACE and GPS. Geophys Res Lett. 10.1029/2009GL038718

[CR52] van Dam T, Blewitt G, Heflin MB (1994) Atmospheric pressure loading effects on global positioning system coordinate determinations. J Geophys Res Solid Earth 99(B12):23939–23950. 10.1029/94JB02122

[CR53] van Dam T, Wahr J, Chao Y, Leuliette E (1997) Predictions of crustal deformation and of geoid and sea-level variability caused by oceanic and atmospheric loading. Geophys J Int 129(3):507–517. 10.1111/j.1365-246X.1997.tb04490.x

[CR54] van Dam T, Wahr J, Milly PCD, Shmakin AB, Blewitt G, Lavallée D, Larson KM (2001) Crustal displacements due to continental water loading. Geophys Res Lett 28(4):651–654. 10.1029/2000GL012120

[CR55] van den Oord A, Dieleman S, Zen H, Simonyan K, Vinyals O, Graves A, Kalchbrenner N, Senior A, Kavukcuoglu K (2016) WaveNet: a generative model for raw audio. 10.48550/arXiv.1609.03499

[CR56] Van Der Knijff JM, Younis J, De Roo APJ (2010) LISFLOOD: a GIS-based distributed model for river basin scale water balance and flood simulation. Int J Geogr Inf Sci 24(2):189–212. 10.1080/13658810802549154

[CR57] Virtanen P, Gommers R, Oliphant TE, Haberland M, Reddy T, Cournapeau D, Burovski E, Peterson P, Weckesser W, Bright J, van der Walt SJ, Brett M, Wilson J, Millman KJ, Mayorov N, Nelson ARJ, Jones E, Kern R, Larson E, Carey CJ, Polat İ, Feng Y, Moore EW, VanderPlas J, Laxalde D, Perktold J, Cimrman R, Henriksen I, Quintero EA, Harris CR, Archibald AM, Ribeiro AH, Pedregosa F, van Mulbregt P, SciPy 1.0 Contributors (2020) SciPy 1.0: fundamental algorithms for scientific computing in Python. Nat Methods 17:261–272. 10.1038/s41592-019-0686-2 32015543 10.1038/s41592-019-0686-2 PMC7056644

[CR58] Wang K, Hu Y, Jiangheng H (2012) Deformation cycles of subduction earthquakes in a viscoelastic Earth. Nature 484:327–332. 10.1038/nature11032 22517160 10.1038/nature11032

[CR59] Waskom ML (2021) seaborn: statistical data visualization. J Open Source Softw 6(60):3021. 10.21105/joss.03021

[CR60] Williams SDP, Penna NT (2011) Non-tidal ocean loading effects on geodetic GPS heights. Geophys Res Lett 38(9):L09314. 10.1029/2011GL046940

[CR61] Xu K, Ba J, Kiros R, Cho K, Courville A, Salakhudinov R, Zemel R, Bengio Y (2015) Show, attend and tell: neural image caption generation with visual attention. In: Bach F, Blei D (eds) Proceedings of the 32nd International Conference on Machine Learning, Proceedings of Machine Learning Research, vol 37. PMLR, Lille, France, pp 2048–2057, 10.48550/arXiv.1502.03044.

[CR62] Yu F, Koltun V (2016) Multi-scale context aggregation by dilated convolutions. In: Bengio Y, LeCun Y (eds) 4th International Conference on Learning Representations, ICLR 2016, San Juan, Puerto Rico, May 2-4, 2016, Conference Track Proceedings, 10.48550/arXiv.1511.07122

