# Supplementary material for: Global data-driven predictions of seasonal non-tectonic signals in vertical GNSS displacement time series from non-tidal surface loading data
Source: Earth Planets Space. 2026 Feb 19;78(1):56. doi: 10.1186/s40623-026-02385-z (PMC13021762; doi:10.1186/s40623-026-02385-z)
Supplement: Supplementary file 1 — Supplementary Material 1. In the supplementary document, we include additional figures that support the main text and include a supplementary text describing the external GNSS data from the IGS repro3 campaign (Rebischung et al. 2024) and the OS LISFLOOD model (Jensen et al. 2025) as an alternative for the hydrological non-tidal loading component used in the analysis in Sect. 5. [file 40623_2026_2385_MOESM1_ESM.pdf]

Supplementary Information for: Global Data-Driven Predictions of Seasonal Non-Tectonic Signals in Vertical GNSS Displacement Time Series from Non-Tidal Surface Loading Data

Kaan Çökerim[1*], Henryk Dobslaw[2], Kyriakos Balidakis[2,3], Laura Jensen[2], Carlos Peña[1,4], Jonathan Bedford[1]

[1*]Tectonic Geodesy Working Group, Institute of Geosciences, Ruhr University Bochum, Bochum, Germany.
[2]Section 1.3. Earth System Modeling, GFZ Helmholtz Centre for Geosciences, Potsdam, Germany.
[3]Federal Agency for Cartography and Geodesy (BKG), Frankfurt am Main,Germany.
[4]University of Potsdam, Potsdam, Germany.

*Corresponding author(s). E-mail(s): kaan.coekerim@ruhr-uni-bochum.de;

Contents of this file

1. Text S1
2. Figures S1 to S13

S1 External Validation and Comparative Analysis of TCN

S1.1 Vertical GNSS Displacements from the IGS repro3 campaign

As an alternative GNSS data source, we utilize coordinate time-series from the combined solution of the extension of the third reprocessing campaign of the International GNSS Service (IGS repro3). The extension includes data from 1994-01-02 to 2022-11-26, 1,060 days more than the IGS contribution to the realization of the latest international terrestrial reference frame, ITRF2020 (Altamimi et al. 2023). We built the coordinate time series employing the combination of daily terrestrial reference frame solutions provided by ten analysis centers carried out by IGS, acquired by a globally distributed tracking network and utilizing the latest reduction models and updated methodologies (http://acc.igs.org/repro3/repro3.html). Unlike time series retrieved from NGL that were obtained by the Precise Point Positioning (PPP) method (Zumberge et al. 1997), the analysis that yielded the IGS repro3 contributions involves network solutions where station coordinates for polyhedra featuring hundreds of nodes were estimated together with parameters that describe Earth rotation and satellite orbits. Moreover, unlike the NGL product, the IGS repro3 offers the highest temporal consistency. Since non-tidal loading was reduced neither at the observation equation, normal-equation, nor parameter level we expect signatures of mass redistribution within Earth's fluid envelope similar to those featured in the NGL product. We refer the interested reader to Rebischung et al. (2024) for further details on the preparation of the combined IGS repro3 solution.

From more than 1,900 stations available within IGS repro3, in this work, we focused on stations that feature within the latest IGS core network, 514. In our analysis we refer to the data as the repro3 data.

S1.2 Experimental Hydrospheric Loading from OS LISFLOOD

As an additional data source to assess our TCN approach, we utilize a recent experiment carried out with the global grid-based hydrological and channel-routing model OS LISFLOOD (Van Der Knijff et al. 2010), which might eventually replace the Land Surface Discharge Model (LSDM) (Dill 2008; Dill et al. 2018) as the terrestrial water storage contribution to the ESMGFZ loading product. OS LISFLOOD has been forced by ERA5 atmospheric fields in a global configuration with 0.05° horizontal resolution and 24-hour temporal resolution. Compared to LSDM, OS LISFLOOD features more accurate evaporation rates in the tropics as well as in arid climates since LSDM-derived potential evaporation stems from 2 m temperature data only. Due to its higher spatial resolution, the OS LISFLOOD implementation simulates riverflow more accurately. Furthermore, it considers more lakes and reservoirs than LSDM. Evaluation results available so far suggest that OS LISFLOOD achieves a more realistic representation of Terrestrial Water Storage variations, especially at interannual time scales. Using the ESMGFZ loading products with OS LISFLOOD as the hydrological component enables us to compare the TCN prediction to state-of-the-art surface loading models and its potential utilization for further developments in numerical Earth system modeling. In the following, we will refer the new total surface loading from OS LISFLOOD + NTAL + NTOL as LISFLOOD NTL or simply ad the OS LISFLOOD model. The hydrological loading time series derived from OS LISFLOOD are currently provided to us for the 514 IGS core stations and not as a global grid. Due to this limited availability at few sparsely distributed stations we currently do not consider OS LISFLOOD as a potential replacement of the LSDM-based HYDL for training the TCN. However, it would be beneficial to test OS LISFLOOD as an alternative hydrological loading input feature to HYDL once it is fully operational and available as a global grid dataset like the other ESMGFZ products.

S1.3 Comparison of TCN predictions to External Data from IGS core repro3 and OS LISFLOOD Loading Model

It is important to note that all results obtained using the NGL hold-out dataset are influenced by both the processing applied by NGL to derive the GNSS displacement time series and by our decomposition process using trajectory modeling to generate an a priori non-tectonic signal dataset. To robustly assess the performance of our TCN model (that was trained on NGL data) in predicting non-tectonic GNSS displacements, we incorporated an external GNSS displacement time series dataset from the IGS core repro3 network. We clearly note that we do not retrain the TCN on the repro3 data but only evaluate the TCN (trained with NGL data) predictions against the repro3 data. As described above in Section S1.2, the repro3 data are network solutions obtained by merging the solutions of multiple analysis centres and satellite constellations while the NGL data are PPP solutions derived from the GPS constellation. As noted by Gómez et al. (2022), even the seasonal non-tectonic signal can vary between GNSS solution depending on the processing technique. Hence, using the IGS repro3 dataset, which is collected and processed independently from the NGL records, provides several advantages. First, it offers a stringent test of model robustness by exposing the TCN to different noise characteristics, data processing pipelines, and environmental conditions inherent in the IGS repro3 observations. Second, while we do compare the TCN predictions on the IGS repro3 data with the hold-out testing data from the NGL dataset, the inclusion of an entirely external dataset minimizes the chance that any observed performance improvements are due to overfitting on the internal characteristics of the NGL data. Hence, this experiment using the external repro3 data tests the TCN's ability to generalize and investigate whether it overfits the random noise or positioning errors in the NGL dataset.

In addition to the ESMGFZ NTL model, we also use another numerical non-tidal model as an additional benchmark which uses OS LISFLOOD as the hydrological

component while retaining NTAL and NTOL from ESMGFZ as the atmospheric and oceanic components. The OS LISFLOOD numerical model is designed to better capture the hydrological component of the non-tidal loading signal and is expected to produce predictions that are closer to the high-quality IGS repro3 data. The LISFLOOD NTL model is published as Jensen et al. (2025).

References

Altamimi, Z., Rebischung, P., Collilieux, X., Métivier, L., Chanard, K.: ITRF2020: an augmented reference frame refining the modeling of nonlinear station motions. Journal of Geodesy **97**(5) (2023) https://doi.org/10.1007/s00190-023-01738-w

Dill, R.: Hydrological model LSDM for operational Earth rotation and gravity field variations. Publisher: Deutsches GeoForschungsZentrum GFZ, 1–35 (2008) https://doi.org/10.2312/GFZ.b103-08095

Dill, R., Klemann, V., Dobslaw, H.: Relocation of River Storage From Global Hydrological Models to Georeferenced River Channels for Improved Load-Induced Surface Displacements. Journal of Geophysical Research: Solid Earth **123**(8), 7151–7164 (2018) https://doi.org/10.1029/2018JB016141

Gómez, D.D., Bevis, M.G., Caccamise, D.J.: Maximizing the consistency between regional and global reference frames utilizing inheritance of seasonal displacement parameters. Journal of Geodesy **96**, 9 (2022) https://doi.org/10.1007/s00190-022-01594-0

Jensen, L., Dill, R., Balidakis, K., Grimaldi, S., Salamon, P., Dobslaw, H.: Global 0.05° water storage simulations with the OS LISFLOOD hydrological model for geodetic applications. Geophysical Journal International **241**(3), 1840–1852 (2025) https://doi.org/10.1093/gji/ggaf129

Rebischung, P., Altamimi, Z., Métivier, L., Collilieux, X., Gobron, K., Chanard, K.: Analysis of the IGS contribution to ITRF2020. Journal of Geodesy **98**(6) (2024) https://doi.org/10.1007/s00190-024-01870-1

Van Der Knijff, J.M., Younis, J., De Roo, A.P.J.: LISFLOOD: a GIS-based distributed model for river basin scale water balance and flood simulation. International Journal of Geographical Information Science **24**(2), 189–212 (2010) https://doi.org/10.1080/13658810802549154

Zumberge, J.F., Heflin, M.B., Jefferson, D.C., Watkins, M.M., Webb, F.H.: Precise point positioning for the efficient and robust analysis of GPS data from large networks. Journal of Geophysical Research: Solid Earth **102**(B3), 5005–5017 (1997) https://doi.org/10.1029/96JB03860 https://agupubs.onlinelibrary.wiley.com/doi/pdf/10.1029/96JB03860

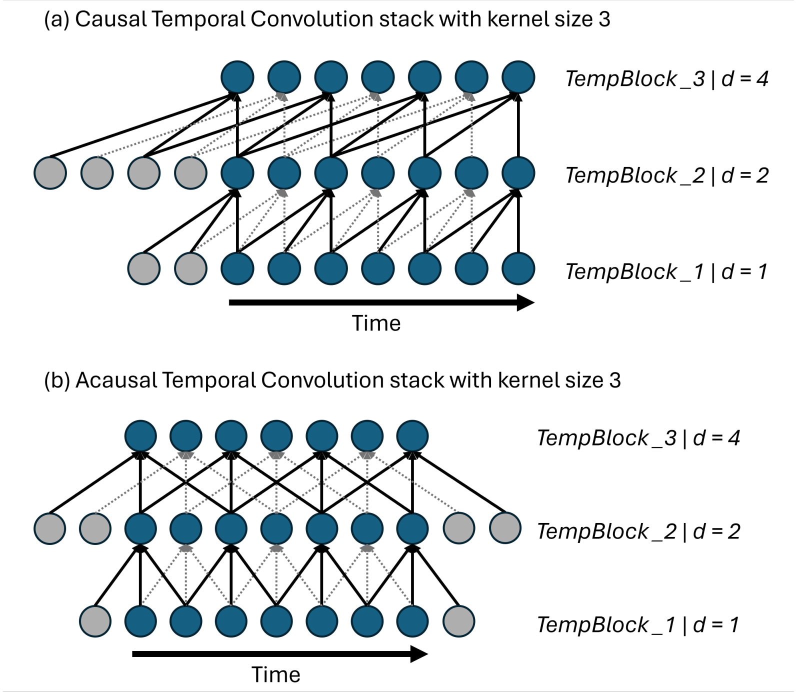

Fig. S1 Dilated convolution stack illustrating causal and acausal padding and receptive fields. The sketch shows a stack of three dilated convolutions with kernel size three and exponentially increasing dilation factor d between layers. Each layer represents a Temporal Convolution Block. While blue dots are the actual time series samples in temporal order from left to right, the gray dots represent zero-padded samples. The zero-padding is only applied on the left side of the time series for the causal case in (a) and equally applied to both sides of the time series in the acausal case depicted in (b). Likewise, the convolution kernel only extends to present and past samples in the causal case (a) but also to future sample in the acausal case (b).

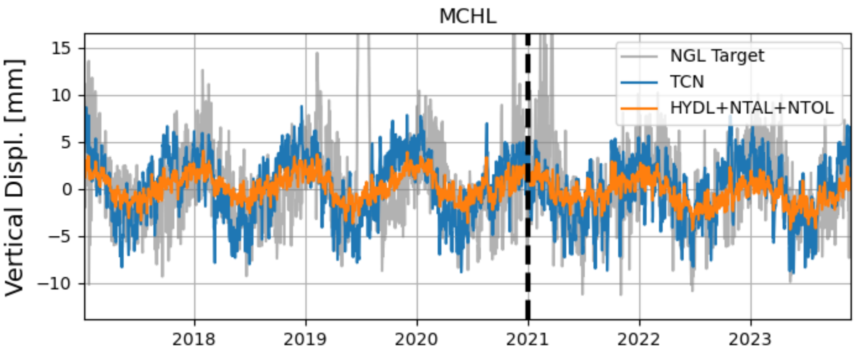

Fig. S2 TCN predictions over many seasonal cycles at station MCHL in Eastern Australia. Predictions over the validation period (2017–2021) and the subsequent hold-out phase to illustrate performance over an extended interval. The TCN prediction is shown in blue and the non-tectonic GNSS target in gray. The ESMGFZ NTL time series (HYDL+NTOL+NTAL) is overlaid in orange for comparison.

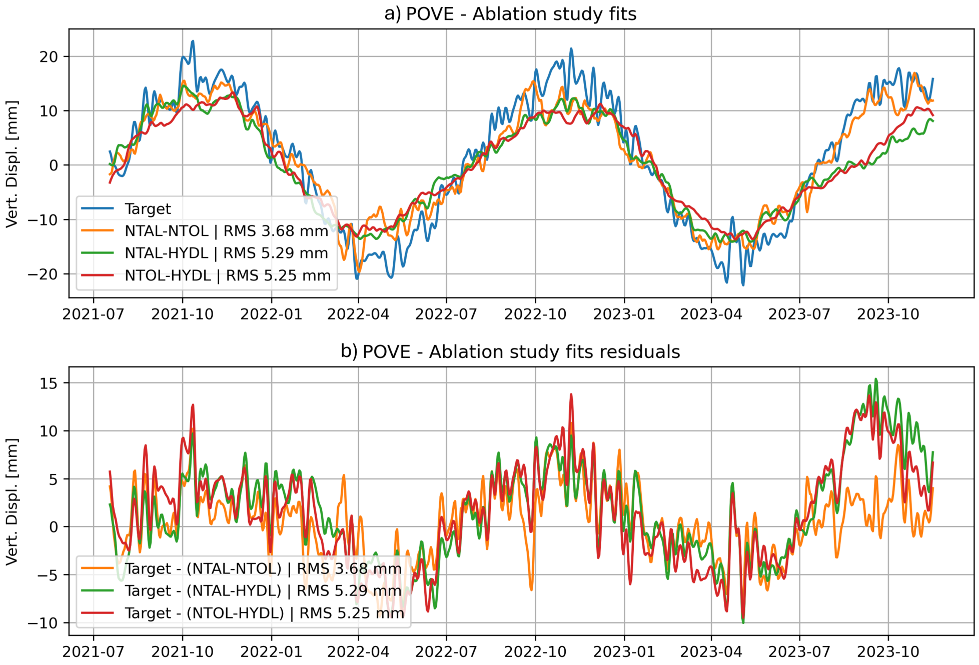

Fig. S3 Time series predictions and target at POVE for the ablation models. (a) Time series at POVE showing the NGL target (blue), and ablation study TCN models with NTAL and NTOL (orange), NTAL and HYDL (green) and NTOL and HYDL (red) as input features with RMS values wrt. the NGL targets. (b) Residual time series between the NGL target and the respective ablation models in same colors as above.

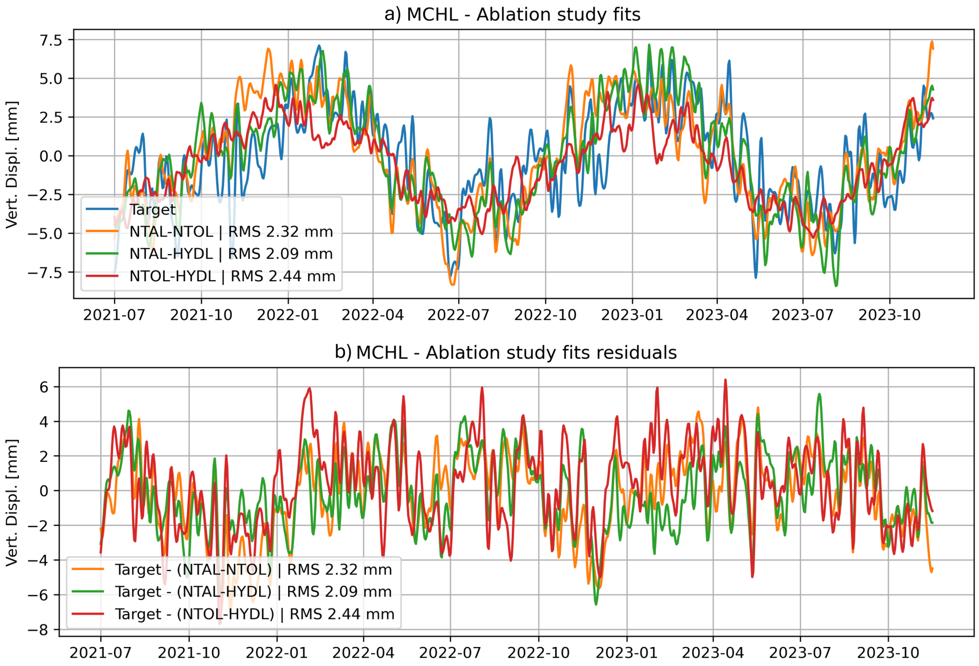

Fig. S4 Time series predictions and target at MCHL for the ablation models. (a) Time series at MCHL showing the NGL target (blue), and ablation study TCN models with NTAL and NTOL (orange), NTAL and HYDL (green) and NTOL and HYDL (red) as input features with RMS values wrt. the NGL targets. (b) Residual time series between the NGL target and the respective ablation models in same colors as above.

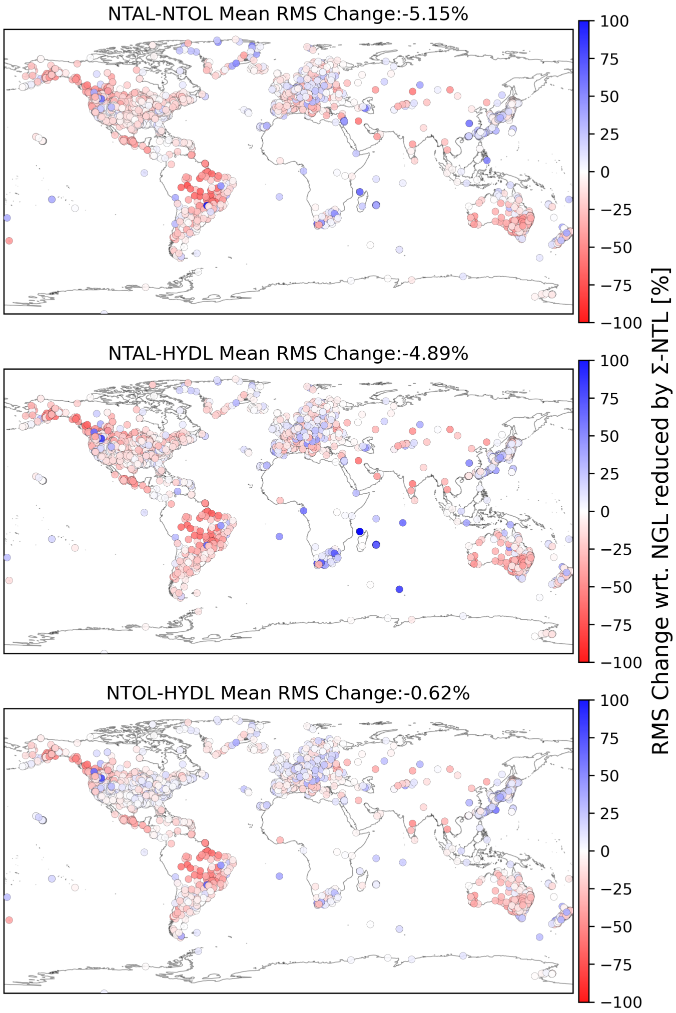

Fig. S5 RMS differences for ablation models relative to ESMGFZ NTL-reduced NGL targets. RMS changes of the TCN-reduced NGL targets relative to the ESMGFZ NTL (Σ-NTL)-reduced NGL targets across all stations. Results are shown for models using (a) NTAL+NTOL, (b) NTAL+HYDL, and (c) NTOL+HYDL as inputs.

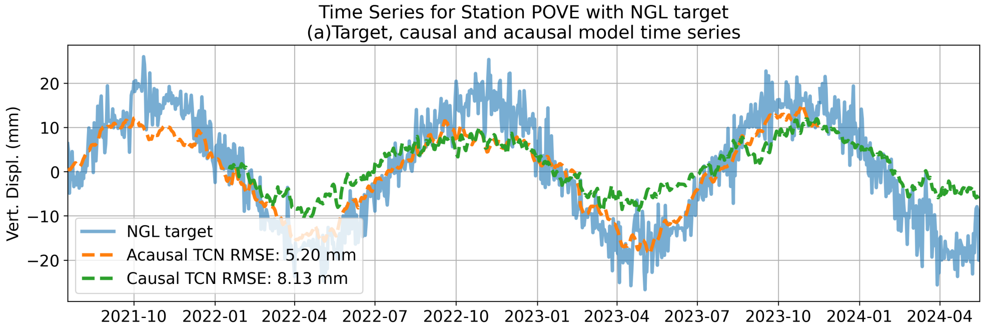

Fig. S6 Causal and acausal TCN predictions at station POVE compared with the NGL target time series. The time series predicted from the causal model is shown in green, the acausal model predictions in orange and the the NGL target time series in blue.

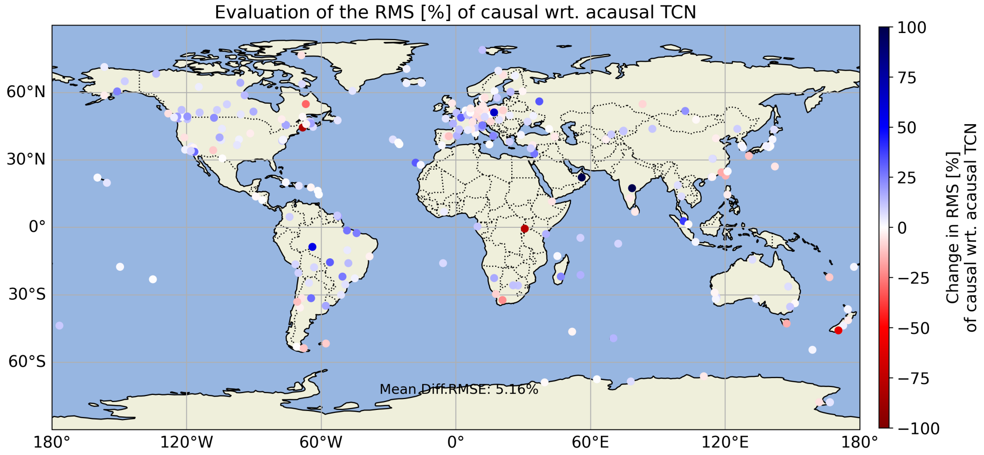

Fig. S7 RMS difference between causal and acausal TCN predictions. The difference is shown at the NGL stations that are also part of the IGS core network.

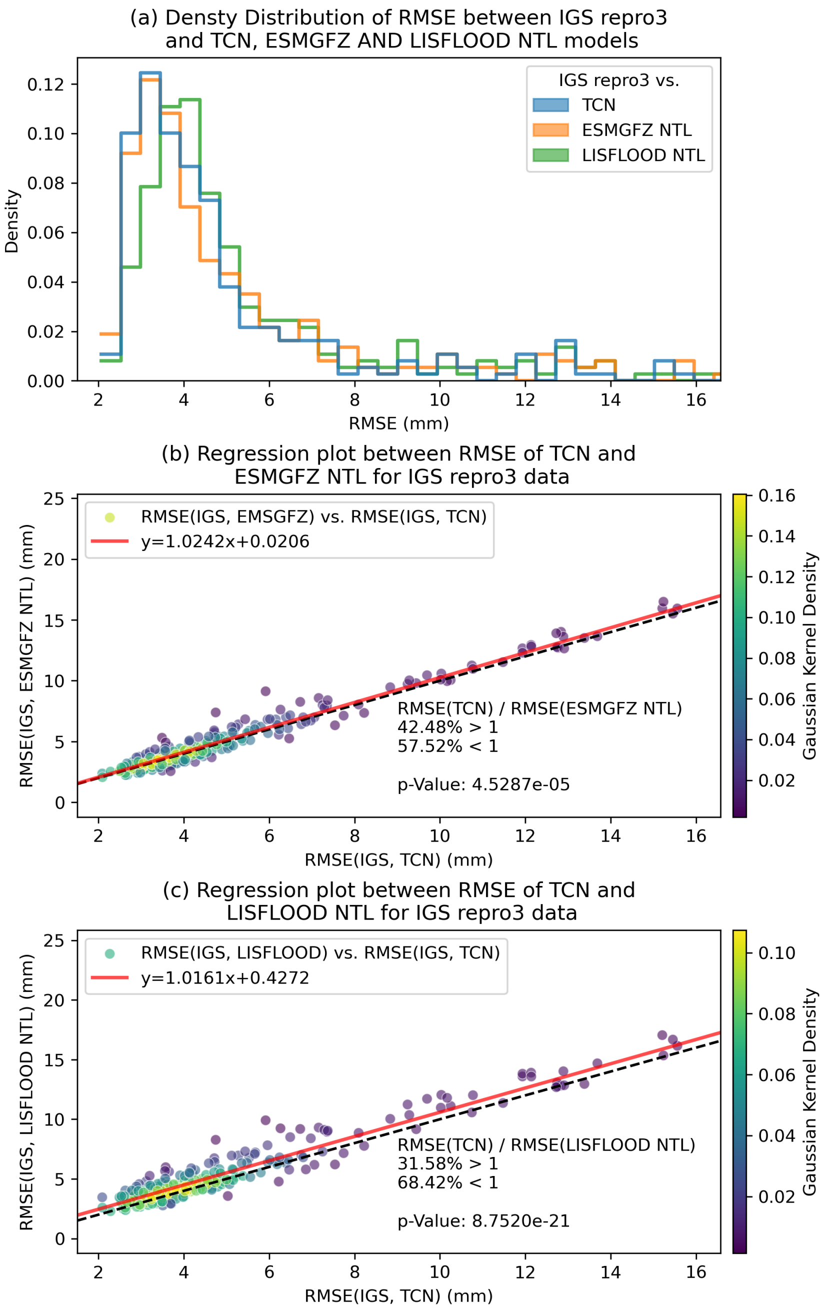

Fig. S8 RMSE statistics of the TCN predictions, ESMGFZ NTL and LISFLOOD models w.r.t. the IGS repro3 targets. (a) Density histograms for the RMSE between the non-tectonic IGS repro3 time series and TCN (blue), ESMGFZ NTL (orange) and LISFLOOD NTL (green). (b) and (c) show a scatter plot of the RMSE between repro3 and TCN on the x-axis and the RMSE between repro3 and ESMGFZ and LISFLOOD respectively. Blue lines in (b) and (c) are the linear region fit through the point cloud and the black dashed line represents the 1:1 ideal model line. Colors in (b) and (c) indicate gaussian kernel density estimate for that point.

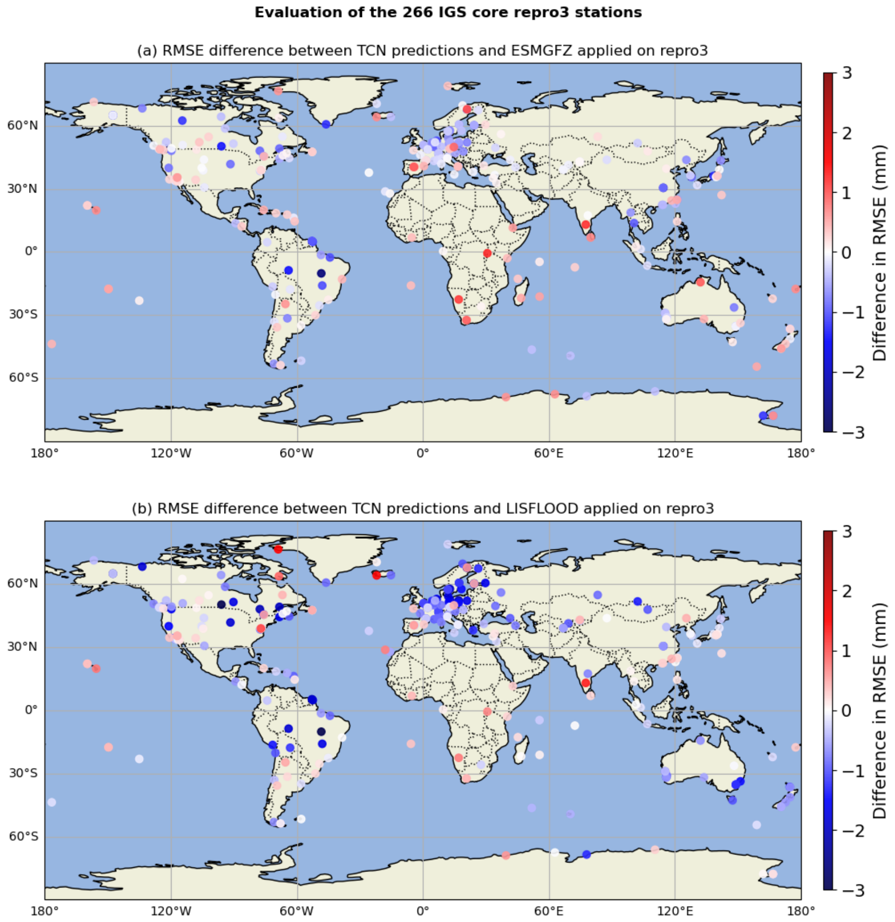

Fig. S9 RMSE differences between TCN and loading models for the IGS repro3 stations. Station-by-station RMSE differences between the TCN predictions and the numeric loading models—(a) ESMGFZ NTL and (b) LISFLOOD—for 266 common stations in the IGS repro3 and NGL datasets. Negative values (blue) indicate improved performance of the TCN relative to the respective loading model, while positive values (red) indicate decreased performance. The color scale shows $\mathrm{RMSE}(\mathrm{repro3}, \mathrm{TCN}) - \mathrm{RMSE}(\mathrm{repro3}, \mathrm{loading\ model})$.

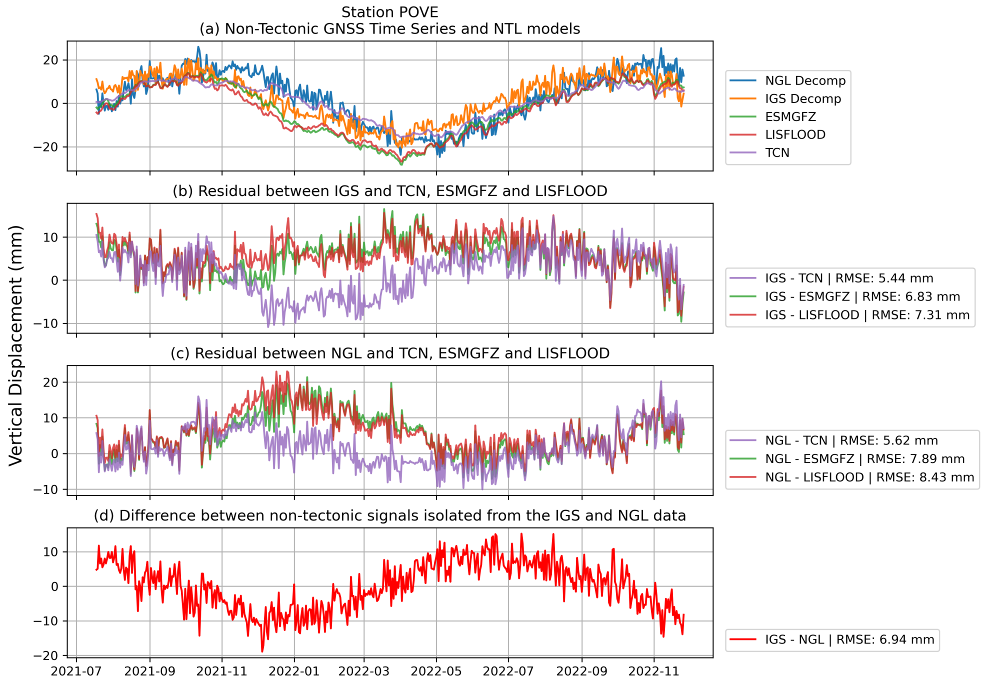

Fig. S10 TCN prediction at POVE and comparison to numerical loading models and the IGS repro3 time series. (a) Decomposed non-tectonic GNSS displacement time series at station POVE from the NGL (blue) and IGS repro3 datasets (orange), along with model predictions from the TCN (purple), ESMGFZ NTL (green), and LISFLOOD (red) loading models. (b) and (c) show the residual of repro3 vs. TCN and NGL vs. TCN respectively (purple). (b) also shows the residual between repro3 and ESMGFZ NTL (green) and repro3 vs. LISFLOOD (red). In (c) the residual between NGL vs. ESMGFZ NTL (green) and NGL vs. LISFLOOD (red) are illustrated. (d) Depicts the residual between the non-tectonic IGS repro3 and NGL time series.

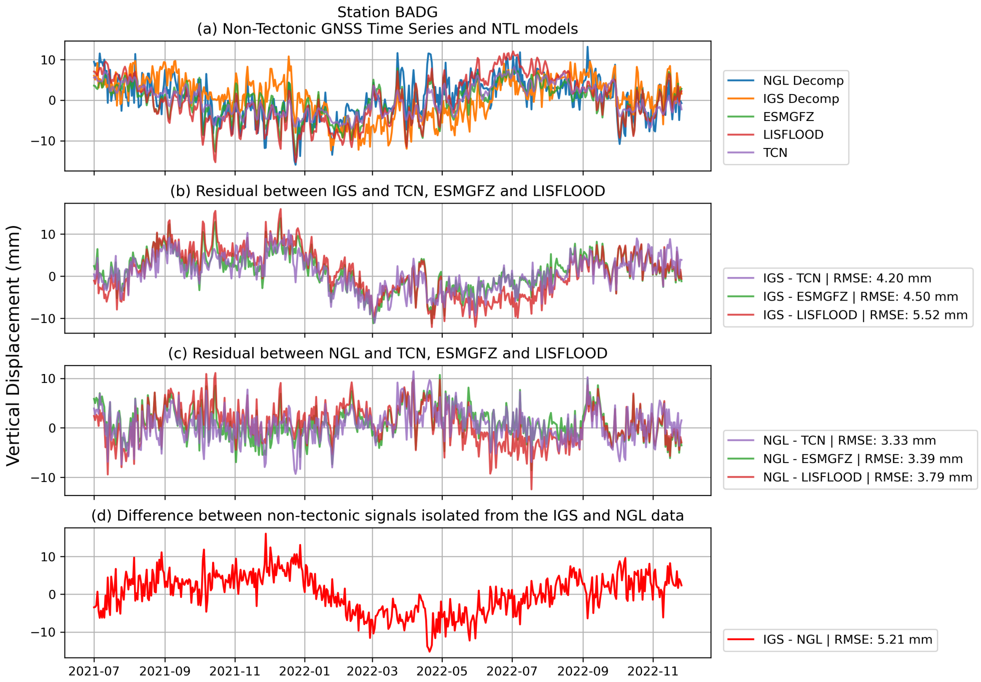

Fig. S11 TCN prediction at BADG and comparison to numerical loading models and the IGS repro3 time series. (a) Decomposed non-tectonic GNSS displacement time series at station BADG from the NGL (blue) and IGS repro3 datasets (orange), along with model predictions from the TCN (purple), ESMGFZ NTL (green), and LISFLOOD (red) loading models. (b) and (c) show the residual of repro3 vs. TCN and NGL vs. TCN respectively (purple). (b) also shows the residual between repro3 and ESMGFZ NTL (green) and repro3 vs. LISFLOOD (red). In (c) the residual between NGL vs. ESMGFZ NTL (green) and NGL vs. LISFLOOD (red) are illustrated. (d) Depicts the residual between the non-tectonic IGS repro3 and NGL time series.

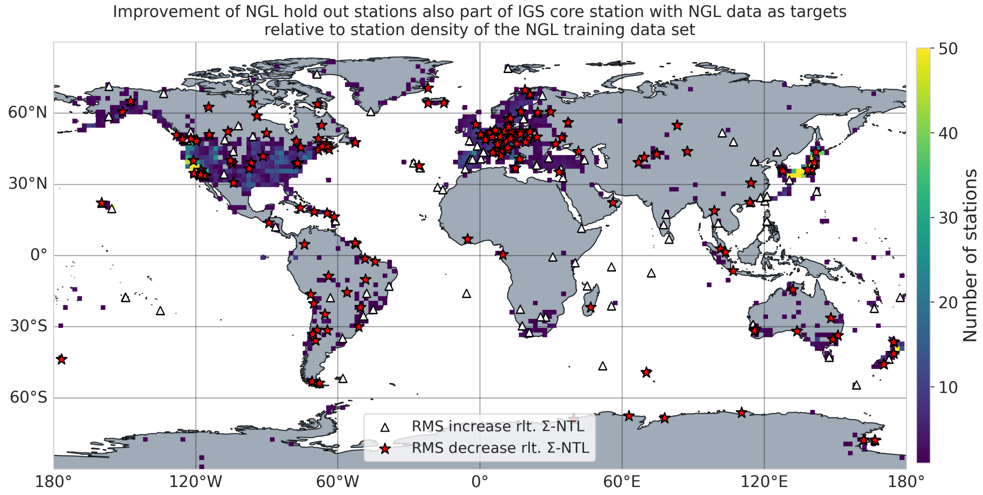

Fig. S12 Spatial density of training stations and performance of the TCN on NGL hold-out targets. Geographic station density showing the number of stations in $2° \times 2°$ bins of the NGL training dataset overlaid by white triangles representing stations exhibiting an increase in RMS and red stars marking a decrease in RMS after subtracting the TCN prediction from the NGL target relative to the RMS value of the Σ-NTL subtracted NGL targets. Here we plot the subset of the NGL dataset that is also part of the IGS core network.

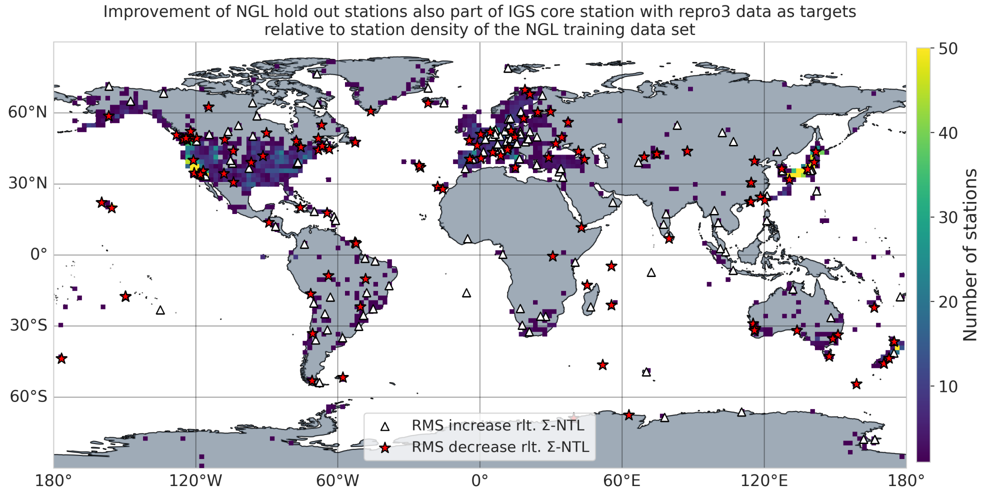

Fig. S13 Spatial density of training stations and performance of the TCN on IGS repro3 targets. Geographic station density showing the number of stations in $2° \times 2°$ bins of the NGL training dataset overlaid by white triangles representing stations exhibiting an increase in RMS and red stars marking a decrease in RMS after subtracting the TCN prediction from the IGS core repro3 target relative to the RMS value of the Σ-NTL subtracted IGS core repro3 targets. Here we plot the IGS repro3 dataset that is part of the NGL dataset.
